# Life’s Essential 8, Genetic Susceptibility, and Metabolic Dysfunction-Associated Steatotic Liver Disease in Chinese Adults

**DOI:** 10.34133/hds.0416

**Published:** 2026-10-19

**Authors:** Shuyao Song, Jiaqi Liu, Ting Wu, Jun Lv, Canqing Yu, Dianjianyi Sun, Lang Pan, Pei Pei, Ling Yang, Yiping Chen, Huaidong Du, Fiona Bragg, Iona Millwood, Robin Walters, Xiaojun Li, Dan Schmidt, Maxim Barnard, Junshi Chen, Zhengming Chen, Liming Li, Yuanjie Pang, China Kadoorie Biobank Collaborative Group

**Affiliations:** ^1^ Department of Epidemiology and Biostatistics, School of Public Health, Peking University, Beijing 100191, China.; ^2^ Peking University Center for Public Health and Epidemic Preparedness and Response, Beijing 100191, China.; ^3^ Key Laboratory of Epidemiology of Major Diseases (Peking University), Ministry of Education, Beijing, China.; ^4^ State Key Laboratory of Vascular Homeostasis and Remodeling, Peking University, Beijing 100191, China.; ^5^ Clinical Trial Service Unit and Epidemiological Studies Unit (CTSU), Nuffield Department of Population Health, University of Oxford, Oxford, UK.; ^6^ Jili Community Health Service, Liuyang, Hunan 410300, China.; ^7^ China National Center for Food Safety Risk Assessment, Beijing 100022, China.

## Abstract

**Background:** Limited evidence exists regarding the association of cardiovascular health (CVH) with metabolic dysfunction-associated steatotic liver disease (MASLD) in Chinese adults, and the influence of genetic risk on this association remains unknown. This study aimed to investigate these associations and discern potential variations based on genetic risk. **Methods:** This cross-sectional study included 17,077 participants from the third resurvey of the China Kadoorie Biobank. CVH was evaluated by Life’s Essential 8 (LE8), with the overall score (0 to 100) calculated as the unweighted average of its 8 component scores and categorized as low (0 to 49), moderate (50 to 79), or high (80 to 100). Polygenic risk score (PRS) for MASLD was generated by meta-analytic approach and large genome-wide association data of liver steatosis and related traits. Genetic risk for MASLD was categorized into low, intermediate, and high based on quintile of metaPRS. The multivariable logistic regression model was used to evaluate the associations of CVH with MASLD and advanced fibrosis in MASLD, subsequently stratified by metaPRS. **Results:** High CVH was associated with over 90% lower risk of MASLD and advanced fibrosis in MASLD, with odds ratios of 0.07 (0.06 to 0.08) and 0.04 (0.02 to 0.05) compared to low CVH, respectively. Interactions of metaPRS were observed for the impacts of LE8 on both prevalent MASLD and advanced fibrosis in MASLD (*P* value for interaction <0.02 and <0.001, respectively), with stronger protective associations among individuals with higher genetic risk. Similar findings were observed when we tentatively expanded LE8 to Life’s Crucial 9 (LC9), incorporating the psychological health component. **Conclusion:** In Chinese adults, adhering to a high CVH was associated with lower odds of prevalent MASLD and advanced fibrosis in MASLD, particularly among individuals at high genetic risk.

## Introduction

Metabolic dysfunction-associated steatotic liver disease (MASLD) is a newly proposed nomenclature of non-alcoholic fatty liver disease (NAFLD), characterized by excess accumulation of hepatic steatosis and cardiometabolic risk factors [1]. MASLD has emerged as a significant global health concern, particularly in China, where an unexpected increase in disease burden has occurred over the past decade [2]. A recent prospective study in China involving 5.7 million adults reported that the prevalence of steatosis and advanced fibrosis was 44.39% and 2.85%, respectively [3]. The overall prevalence of NAFLD is 32.5%, and the incidence is 47.3 cases per 1000 person-years in China [4]. MASLD is associated with progressive fibrosis and cirrhosis, hepatocellular carcinoma, as well as higher risk of all-cause and cardiovascular disease (CVD) mortality.

Lifestyle factors play pivotal roles in the development and progression of MASLD [5]. Life’s Essential 8 (LE8) is an updated and enhanced approach to measuring, monitoring, and modifying cardiovascular health (CVH) proposed by the American Heart Association (AHA) recently. It includes 8 components that are closely related to health behaviors and health factors and allows the generation of a new composite CVH score [6]. Furthermore, given the pivotal role of psychological health in the risk, onset, and recurrence of CVD, the Life’s Crucial 9 (LC9) framework has been proposed as a tentative expansion of LE8 to provide a more integrated assessment of CVH [7]. Recently, prospective cohort studies in China have shown protective associations of LE8 with CVD, supporting the applicability of LE8 for cardiometabolic health in Chinese adults [8,9]. However, there is limited evidence on the impact of CVH on MASLD and advanced fibrosis in the Chinese population, with existing studies largely confined to regional populations (e.g., the Jiading District of Shanghai and the Kailuan community in Tangshan), and thus, systematic evidence from a nationwide large-scale population is lacking [10,11].

In addition to lifestyle, genetic susceptibility also plays an important role in the development of MASLD [12], while polygenic risk scores (PRSs) for NAFLD have been developed in Europeans utilizing variants related to liver steatosis [13–15]. Previous studies mainly constructed unweighted PRS for NAFLD based on genome-wide association studies (GWASs) for single traits, resulting in imprecise risk stratification. Additionally, there is limited application of PRS for MASLD in non-European populations due to the ancestry-specific differences in the frequency, effect sizes, and linkage disequilibrium patterns of genetic variants [16]. Recently, a meta-analytic approach combining large GWAS results for coronary artery disease (CAD) and CAD-related traits in East Asians led to the development of the combined PRS (metaPRS) for CAD in Chinese adults [17,18]. By integrating genetic variants associated with both the primary disease and its related traits, metaPRS captures the shared genetic architecture and pleiotropic effects, thereby enhancing the predictive capacity for multifactorial diseases and demonstrating significant potential for improved risk stratification [19]. As MASLD involves metabolic components [i.e., body mass index (BMI), plasma glucose, blood pressure (BP), and blood lipids] [1], developing metaPRS for MASLD that is tailored to Chinese adults, by utilizing GWAS data of liver steatotic and metabolic-related traits, is promising to enhance risk stratification and help identify individuals benefiting more from lifestyle interventions.

Importantly, exploring the interaction between CVH and genetic susceptibility in relation to the risks of MASLD and advanced fibrosis is crucial for identifying high-risk individuals who may benefit most from lifestyle interventions and for formulating personalized prevention strategies. However, whether genetic risk modifies the associations of CVH with MASLD and its progression remains to be systematically investigated.

Therefore, the primary objectives of our study were (a) to evaluate the associations of CVH, measured by updated LE8 framework, with risks of MASLD and advanced fibrosis in the Chinese population; (b) to construct a genetic risk score for MASLD by incorporating the large genome-wide association data of liver steatosis and liver steatosis-related traits; and (c) to examine the interactions of CVH and genetic risk score on risks of MASLD and advanced fibrosis in MASLD.

## Methods

### Study population

The cross-sectional study involved 17,077 participants attending the third resurvey in the China Kadoorie Biobank (CKB) with genotyping information and without cancer, cirrhosis, or viral hepatitis (Fig. S1). The CKB recruited 512,724 individuals (210,205 men and 302,519 women) aged 30 to 79 years living in 10 regions across China (5 urban and 5 rural) during the baseline survey in 2004–2008. Details of the CKB design, survey methods, and population characteristics have been described elsewhere [20]. After completion of the baseline survey, this project randomly selected ~5% of the individuals who remained in the cohort from each of the 10 study areas. The third resurvey took place between August 2020 and December 2021, involving 25,078 participants [21]. Laptop-based questionnaires were administered by interviewers collecting information on sociodemographic characteristics, smoking and alcohol consumption, diet, physical activity, and medical history. A range of physical measurements were conducted including height, weight, waist circumference, BP, and transient elastography for liver assessment, and bio-samples (blood, urine, and feces) were collected by trained technicians with standard protocols. The CKB study was conducted in accordance with the Declaration of Helsinki, with approval from the Ethical Review Committee of the Chinese Center for Disease Control and Prevention (Beijing, China) and the Oxford Tropical Research Ethics Committee, University of Oxford (UK). All participants provided written informed consent.

### CVH assessment

CVH was measured using AHA’s LE8 algorithm, including 4 health behaviors (diet, physical activity, smoking, and sleep) and 4 health factors (BMI, blood lipids, blood glucose, and BP) [6]. Detailed measurements of LE8 metrics in CKB were outlined in Supplementary Methods 1. Previous validation studies in CKB showed good validity and reliability of questionnaire-based assessment of health behaviors, and health factors were recorded by trained technicians using calibrated instruments with standard protocols (Supplementary Methods 2 to 7). Each of the 8 CVH components was scored on a scale of 0 to 100 points, and then the aggregate score was scaled from 0 to 100 points, calculated as the unweighted average of all 8 component metric scores. To develop and evaluate integrated models of CVH that encompass the management of psychological health, we tentatively expanded the LE8 framework, with the addition of psychological health components, the LC9 (Supplementary Methods 8) [7]. The overall CVH score was categorized as high (80 to 100), moderate (50 to 79), and low (0 to 49) as recommended by AHA [6].

### Genotyping and generation of metaPRS

Of the 25,078 participants attending the third resurvey, 17,972 randomly selected participants were genotyped using a custom-designed 800K-single-nucleotide variation (SNV) array (Axiom, Affymetrix) with imputation to 1000 Genomes Phase 3. Of all participants attending the third resurvey, baseline characteristics were similar in participants with and without genotyping information (Table S1).

To construct a combined polygenetic risk score (metaPRS) for MASLD, 8 PRS for specific traits were separately constructed, including NAFLD and 7 NAFLD-related traits [type 2 diabetes (T2D), BMI, total cholesterol (TC), high-density lipoprotein cholesterol (HDL-C), triglycerides (TGs), low density lipoprotein-cholesterol (LDL-C), and BP; Fig. S2]. Figure S3 shows the correlations between the NAFLD PRS and each trait-specific PRS. The NAFLD PRS in this study incorporated genetic variants demonstrating genome-wide significant associations by previous GWAS studies (Table S2) [14,15,22–25]. Each PRS for NAFLD-related traits was established based on previously constructed PRS developed in prospective Chinese cohort studies (Table S3) [17,18]. Notably, these source GWAS, which provided both the genetic variants and their corresponding effect sizes, were independent of our study population with no sample overlap. Eight trait-specific PRS were separately constructed by summing the number of corresponding risk alleles (0, 1, or 2) for each individual, weighted by the effect size of variants derived from these source GWAS. The metaPRS was then generated by integrating the 8 individual PRS using elastic-net logistic regression [17,18,26]. We also categorized the metaPRS into low (lowest quintile), intermediate (quintiles 2 to 4), and high (highest quintile) genetic risk based on quintile distributions of metaPRS [18]. Detailed information on the genetic variants and their corresponding weights for both the trait-specific PRS and the metaPRS, along with further construction details, are provided in Table S4 and Supplementary Methods 9.

### Outcomes

Trained and certified doctors performed vibration-controlled transient elastography (VCTE) using FibroTouch FT-100 (Hisky, Wuxi, China) in accordance with the standard protocol. Fat attenuation parameter (FAP) and liver stiffness measurement (LSM) were derived to assess liver steatosis and fibrosis, respectively. For a VCTE examination to be considered eligible, a success rate of >60% should be achieved with 10 successful measurements.

According to the latest consensus [1], MASLD was diagnosed based on liver steatosis (FAP ≥ 244 dB/m) [3], and the presence of any of the following 5 cardiometabolic conditions: (a) BMI ≥ 23 kg/m^2^ or waist circumference (WC) ≥ 90/80 cm for men/women, (b) fasting plasma glucose (FPG) ≥ 100 mg/dl or random plasma glucose (RPG) ≥ 140 mg/dl or T2D or treatment for T2D, (c) BP ≥130/85 mmHg or specific antihypertensive drug treatment, (d) plasma TGs ≥150 mg/dl or lipid-lowering treatment, and (e) plasma HDL-C ≤ 40/50 mg/dl for men/women or lipid-lowering treatment. The presence of liver steatosis and fibrosis was defined as follows: FAP values ≥244 dB/m, ≥269 dB/m, and 296 dB/m indicated mild, moderate, and severe liver steatosis (grade ≥S1, ≥S2, and S3, respectively). Liver fibrosis grade is ≥F2 (significant fibrosis and above) if LSM ≥7.85 kPa, ≥F3 (advanced fibrosis and above) if LSM ≥10.0 kPa, and F4 (cirrhosis) if LSM ≥13.5 kPa [3]. The cutoff values for liver steatosis and fibrosis were established in large-scale cross-sectional surveys in Chinese adults [3,27,28].

The vital status of each participant was determined periodically through the China CDC’s Disease Surveillance Points (DSP) system and the national health insurance system, supplemented by regular checks against local residential and health insurance records and by annual active confirmation through street committees or village administrators [29]. Of the 17,077 participants in this study, 451 deaths occurred until 2023 December 1.

### Statistical analyses

Because of the rigorous quality control of the CKB study, there were no missing data for any core variables in this analysis, including LE8 metrics, MASLD diagnostic components, and covariates. The associations of CVH and PRS with MASLD, advanced fibrosis in MASLD, and all-cause mortality were quantified using multivariable logistic regression models. For CVH, the model was adjusted for age, sex, region, marital status, household income, and education, which were selected based on previous literature [30–32]. The association of each individual component of the CVH with MASLD and advanced fibrosis in MASLD was also examined, with all 8 metrics of LE8 simultaneously included in the model. For PRS, the model was adjusted for age, sex, region, and 11 national principal components, which were selected to effectively account for population stratification and capture the geographic genetic structure across the diverse study regions of the CKB study [33].

To investigate whether the genetic predisposition could be offset by CVH, a stratified analysis was conducted by metaPRS to investigate associations of CVH with MASLD and advanced fibrosis in MASLD among adults in different subgroups of genetic risk. An interaction between LE8 and metaPRS was tested by adding a cross-product of LE8 and metaPRS into the models using the likelihood ratio test. Restricted cubic splines with 5 knots (at the 5th, 25th, 50th, 75th, and 95th percentiles) were empirically used to assess the dose–response relationship of CVH score with MASLD and advanced fibrosis in MASLD. To assess the joint associations, we further classified participants into 9 groups according to CVH categories (low, moderate, and high) and genetic risk categories (low, intermediate, and high) and estimated odds ratios (ORs) for MASLD and advanced fibrosis in MASLD in different groups compared with those with low genetic risk and high CVH.

*P* values were 2-sided, and statistical significance was set at less than 0.05. For the associations of CVH with MASLD and advanced fibrosis, more stringent Bonferroni-adjusted thresholds of 0.05/8 (*P* < 0.00625) for LE8 and 0.05/9 (*P* < 0.00556) for LC9 were applied. Prevalence estimates were calculated as proportions, with 95% confidence intervals (CIs) estimated by the normal approximation to the binomial distribution. Statistical analyses were performed with Stata version 17.0 and R version 4.2.3.

## Results

### Basic characteristics

Of the 17,077 participants included, the mean age at the third resurvey was 65.2 years (SD, 9.2 years) and 63.9% were women. The overall mean CVH score was 61.4 (95% CI, 61.3 to 61.6). Scores were higher among women than men (mean: 63.2 versus 57.3 in urban area; 63.6 versus 58.2 in rural area; Fig. 1). Of all participants, 6.8% met the high CVH criteria, 76.6% were in the moderate CVH category, and 16.5% were in the low CVH category. Participants with a high CVH were younger, more likely to be female, and less likely to be weekly drinkers (Table 1). The distribution of the overall LE8 score and each component of LE8 among the study participants was shown in Fig. S4.

**Fig. 1.**
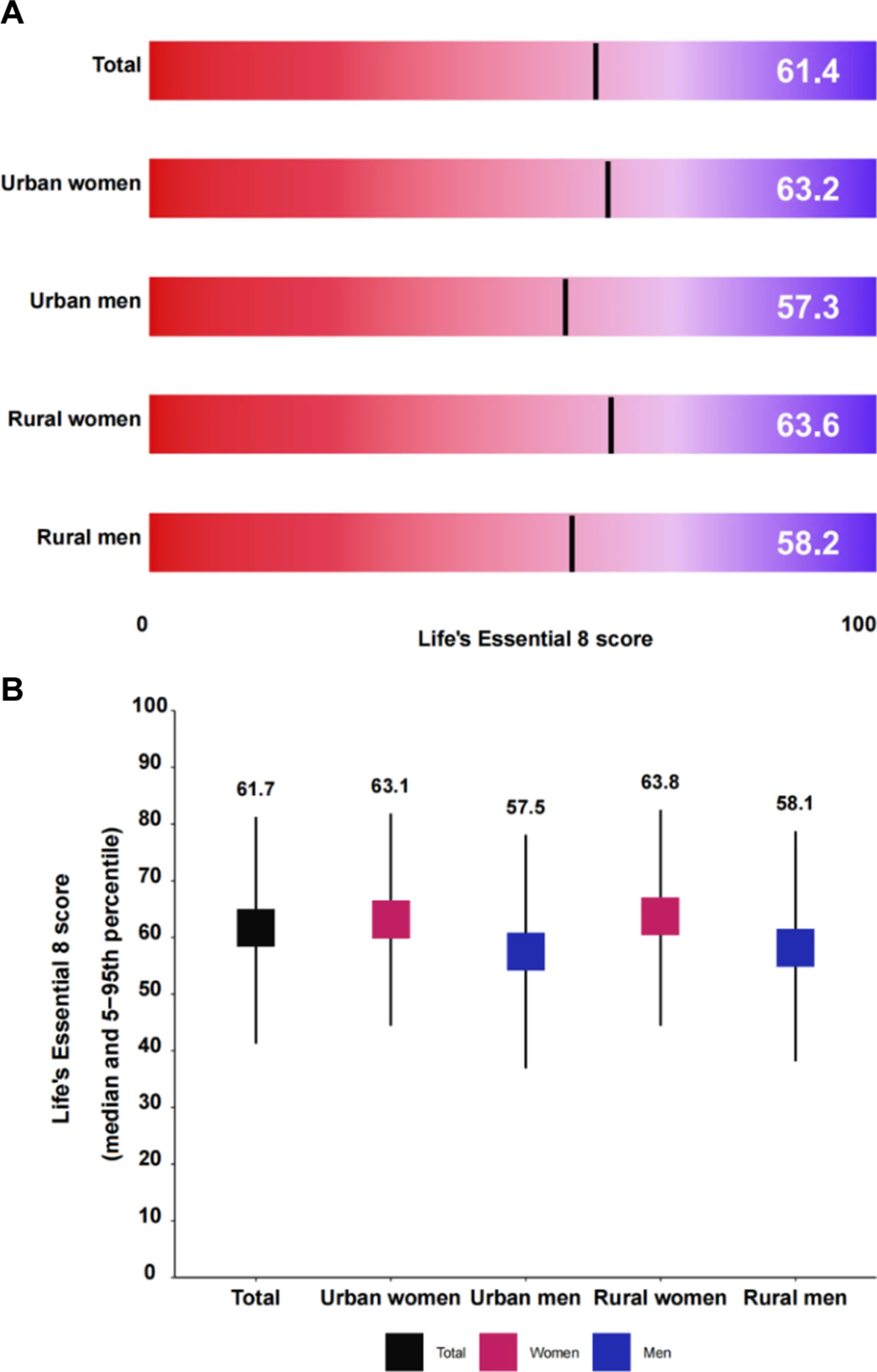
Life’s Essential 8 CVH scores, overall and by region and sex. Scores derived from CKB third resurvey population, 2020 to 2021. (A) Mean (thick black bar). (B) Median (square) and 5th to 95th percentile (black lines). CVH, cardiovascular health.

**Table 1.** Baseline characteristics according to Life’s Essential 8 cardiovascular health status

Variable ^a^	Cardiovascular health			
	Low	Moderate	High	Overall
	(*n* = 2,823)	(*n* = 13,080)	(*n* = 1,174)	(*n* = 17,077)
Age (SD), year	66.6 (8.9)	65.2 (9.2)	61.9 (8.8)	65.23 (9.21)
Female, %	1235 (44.6)	8725 (66.8)	947 (80.5)	10907 (63.9)
**Socioeconomic and lifestyle factors**				
Urban region, %	1015 (35.9)	4417 (33.7)	384 (33.2)	5816 (34.1)
≥9 years of education, %	538 (14.8)	1920 (15.3)	138 (14.8)	2596 (15.2)
Household income ≥35,000 RMB/year, %	2422 (86.0)	11037 (84.4)	978 (82.0)	12533 (73.4)
Ever regular smoking, %
Male	1338 (84.5)	2597 (59.2)	44 (20.2)	3979 (64.7)
Female	76 (5.8)	133 (1.9)	3 (0.4)	212 (2.0)
Weekly drinking, %				
Male	543 (34.2)	1224 (28.0)	33 (15.3)	1800 (29.3)
Female	38 (2.7)	164 (1.9)	18 (2.0)	220 (2.0)
Total physical activity (SD), MET-h/day	11.6 (10.5)	16.1 (14.1)	22.9 (14.9)	15.8 (14.0)
Snoring, %	2005 (71.3)	6443 (49.4)	269 (21.0)	8717 (51.2)
**Blood pressure and anthropometry**				
SBP (SD), mmHg	143.4 (19.6)	131.9 (20.6)	117.2 (14.0)	132.9 (21.1)
RPG (SD), mg/dl	8.1 (3.7)	6.2 (2.2)	5.5 (1.1)	6.5 (2.6)
LDL (SD), mg/dl	3.7 (1.7)	3.3 (1.3)	2.9 (0.9)	3.31 (1.3)
BMI (SD), kg/m^2^	26.6 (3.4)	24.1 (3.3)	21.6 (2.4)	24.4 (3.5)
**Liver VCTE measurement**				
FAP (IQR), dB/m	43.9 (5.5)	63.9 (7.8)	84.1 (3.9)	248.0 (52.0)
LSM (IQR), kPa	41.4 (13.7)	59.9 (14.1)	80.2 (9.6)	6.9 (3.0)
Advanced fibrosis, % ^b^	56.7 (13.8)	81.3 (15.8)	107.0 (10.2)	2256 (13.2)
**CVH**				
LE8 score (SD)	270.0 (47.0)	245.0 (50.0)	224.0 (33.0)	61.4 (12.3)
LE8 score behavior scale (SD)	7.2 (3.5)	6.8 (2.9)	6.6 (2.6)	57.6 (16.8)
LE8 score biological scale (SD)	542 (18.5)	1605 (12.2)	109 (10.1)	78.1 (19.4)
**Prior disease history, %**				
Diabetes	882 (31.2)	1421 (10.7)	50 (4.4)	2367 (13.9)
Ischemic heart disease	354 (11.5)	1061 (8.1)	43 (4.9)	1458 (8.6)
Stroke or TIA	281 (8.9)	767 (5.9)	34 (3.7)	1082 (6.4)
Hypertension	1560 (56.1)	4365 (32.8)	109 (9.9)	6034 (35.4)
Family history of diabetes	500 (17.8)	1714 (13.1)	108 (8.7)	2324 (13.7)
Family history of cancer	654 (23.3)	2886 (22.0)	234 (20.1)	3780 (22.2)
**Medication use, %**				
Statin medication	313 (10.7)	446 (3.3)	4 (0.4)	763 (4.5)
Antihypertensive	1470 (52.7)	3945 (29.7)	89 (8.2)	5504 (32.3)
Diabetes treatment	631 (22.2)	1098 (8.3)	42 (3.7)	1771 (10.4)
**Genetic risk category, %**				
Low	744 (18.0)	2465 (20.0)	140 (21.9)	3349 (19.6)
Intermediate	1664 (58.3)	7897 (59.9)	704 (60.8)	10265 (60.1)
High	415 (23.7)	2718 (20.1)	330 (17.3)	3463 (20.3)

BMI, body mass index; FAP, fat attenuation parameter; LE8, Life’s Essential 8; LSM, liver stiffness measurement; MASLD, metabolic dysfunction-associated steatotic liver disease; MET, metabolic equivalent of the task; SBP, systolic blood pressure; TIA, transient ischemic attack; VCTE, vibration-controlled transient elastography

^a^ Values are means [standard deviation (SD)], median [interquartile range (IQR)], or *n* (%). Results were standardized by age, sex, and region where appropriate.

^b^ Advanced fibrosis is defined as LSM ≥10.0 kPa.

Among all participants, the prevalence was 52.0% (95% CI, 51.3% to 52.8%) for MASLD (*n* = 8,885) and 8.5% (95% CI, 8.1% to 8.9%) for advanced fibrosis in MASLD (*n* = 1,453). The prevalence of MASLD and advanced fibrosis in MASLD in women was higher than that in men (MASLD: 54.8% versus 47.1%; advanced fibrosis in MASLD: 8.6% versus 8.3%). The prevalence of MASLD and liver fibrosis in MASLD with age showed an inverse U-shaped pattern, peaking at 55 to 59 and 70 to 74 years, respectively (Fig. 2). Participants with MASLD were younger, more likely to be women and from urban areas, had lower levels of education, and were more likely to be weekly drinkers (particularly in men; Table S5).

**Fig. 2.**
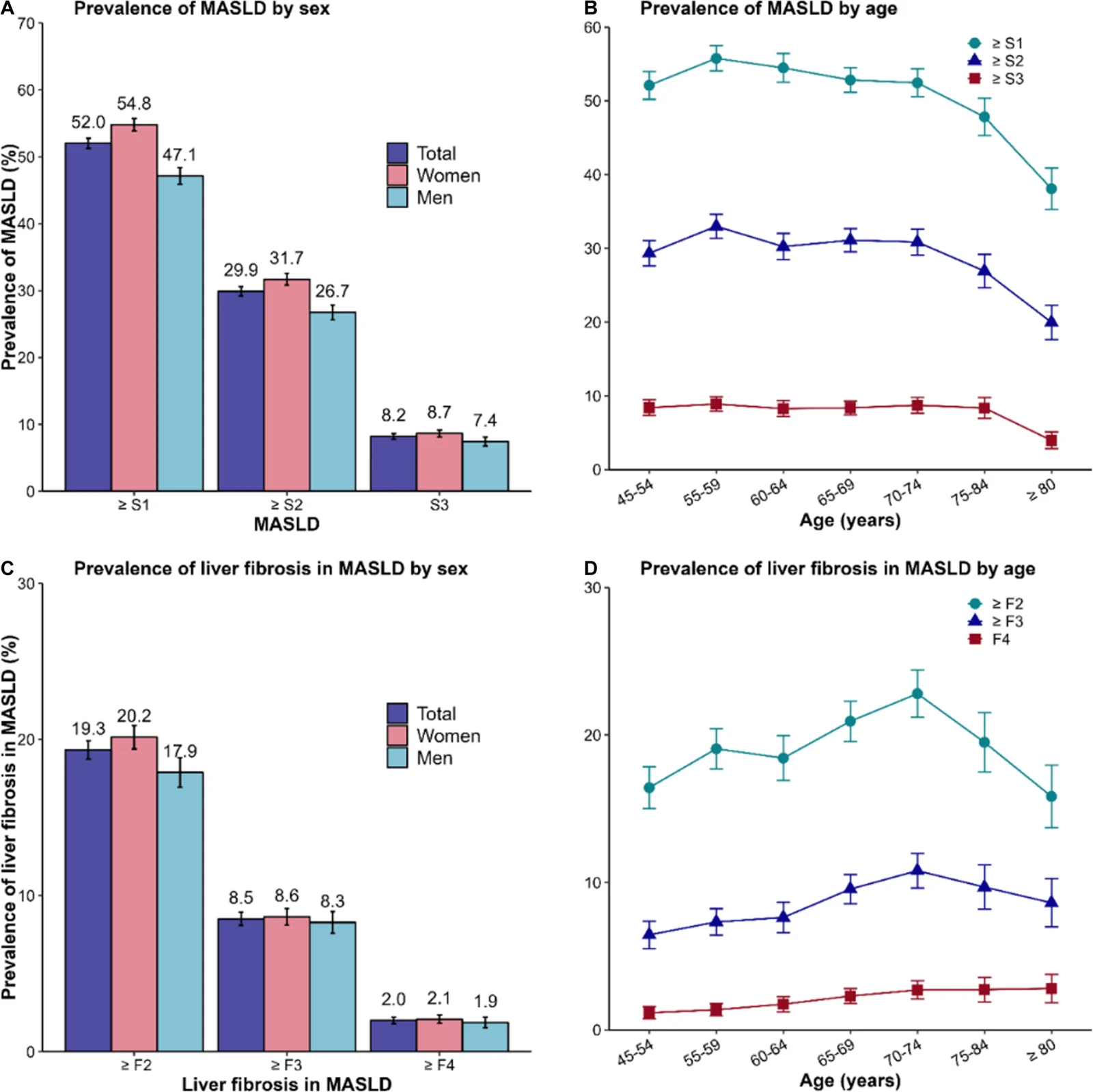
Prevalence of MASLD and advanced fibrosis in MASLD by sex and age. Prevalence of MASLD with different grades of liver steatosis (A) by sex and (B) by age. Prevalence of advanced fibrosis in MASLD with different grades of liver fibrosis (C) by sex and (D) by age. FAP values ≥244, ≥269, and 296 dB/m indicated mild, moderate, and severe liver steatosis (grade ≥S1, ≥S2, and S3, respectively). LSM values ≥7.85, ≥10.0, and 13.5 kPa indicated significant fibrosis, advanced fibrosis, and cirrhosis, respectively (≥F2, ≥F3, and F4, respectively). LE8, Life’s Essential 8; MASLD, metabolic dysfunction-associated steatotic liver; PRS, polygenetic risk score.

### Associations of CVH with MASLD and advanced fibrosis in MASLD

Compared with participants with low CVH, the ORs of MASLD were 0.29 (95% CI, 0.26 to 0.32) for moderate CVH (measured by LE8) and 0.07 (0.06 to 0.08) for high CVH, while the ORs of advanced fibrosis in MASLD were 0.21 (0.18 to 0.24) for moderate CVH and 0.04 (0.02 to 0.05) for high CVH. Each SD increment in LE8 score (12.2 scores) was associated a 55% (OR = 0.45 [0.43 to 0.46]) lower risk of MASLD and 63% (OR = 0.37 [0.34 to 0.39]) lower risk of advanced fibrosis in MASLD (Table 2). Nonlinear associations were not observed for LE8 score with MASLD and advanced fibrosis in MASLD (*P* value for nonlinear > 0.05; Fig. S5). Each SD increment in LE8 was associated with 13%, 14%, and 16% lower risk of mortality in the total population, MASLD, and non-MASLD population, respectively (OR = 0.89 [0.79 to 0.97], 0.86 [0.74 to 1.01], and 0.84 [0.74 to 0.97]; Table S6). When expanding the LE8 framework into LC9, by tentatively adding the psychological health component, similar protective associations were observed between CVH (measured by LC9) and risk of MASLD or advanced fibrosis in MASLD (high versus low CVH: OR = 0.06 [0.05 to 0.09] for MASLD, OR = 0.03 [0.01 to 0.07] for advanced fibrosis in MASLD; Table S7). The associations of each individual component of LE8 and psychological health component with MASLD and advanced fibrosis in MASLD were shown in Fig. S6.

**Table 2.** Associations of LE8 with MASLD and advanced fibrosis in MASLD, overall and by stratum of genetic risk.

	MASLD		Advanced fibrosis in MASLD ^a^	
	OR (95 CI%)	*P* value ^b^	OR (95 CI%)	*P* value ^b^
**CVH category, overall**				
Low CVH	Reference		Reference	
Moderate CVH	0.29 (0.26, 0.32)	<0.001	0.21 (0.18, 0.24)	<0.001
High CVH	0.07 (0.06, 0.08)	<0.001	0.04 (0.02, 0.05)	<0.001
**CVH score, per SD increment**				
Overall	0.45 (0.43, 0.46)	<0.001	0.37 (0.34, 0.39)	<0.001
High genetic risk	0.41 (0.37, 0.45)	<0.001	0.32 (0.27, 0.38)	<0.001
Intermediate genetic risk	0.46 (0.43, 0.48)	<0.001	0.37 (0.34, 0.41)	<0.001
Low genetic risk	0.47 (0.43, 0.51)	<0.001	0.40 (0.34, 0.47)	<0.001
*P* value for interaction ^c^	0.019		<0.001	

CI, confidence interval; CVH, cardiovascular health; LE8, Life’s Essential 8; MASLD, metabolic dysfunction-associated steatotic liver disease; OR, odds ratio; PRS, polygenetic risk score

The multivariable logistics model was used to estimate ORs and 95% CIs, adjusted for age, sex, region, marital status, household income, and education. Analysis was conducted on participants who did not have MASLD as the reference group.

^a^ Advanced fibrosis is defined as LSM ≥10.0 kPa.

^b^ The associations of CVH with MASLD and advanced fibrosis in MASLD were observed when using the Bonferroni-adjusted *P* value threshold of 0.00625 (0.05/8).

^c^ Interactions were tested using a likelihood ratio test to compare 2 linear models, including the interaction term of metaPRS and LE8.

### Associations of polygenetic risk with MASLD and advanced fibrosis in MASLD

The magnitude of association between metaPRS and MASLD was greater than NAFLD PRS or other individual trait-specific PRS (Fig. S7). MetaPRS was a key predictor for MASLD (*R*^2^ = 1.47%, *F* = 254.4) and performed well in risk stratification (Fig. 3 and Fig. S8). For MASLD, the top versus bottom quintile ORs of metaPRS and NAFLD PRS were 2.01 (1.77 to 2.29) and 1.26 (1.14 to 1.39), respectively; for advanced fibrosis in MASLD, the corresponding ORs were 2.24 (1.76 to 2.85) and 1.46 (1.22 to 1.75), respectively (Table S8).

**Fig. 3.**
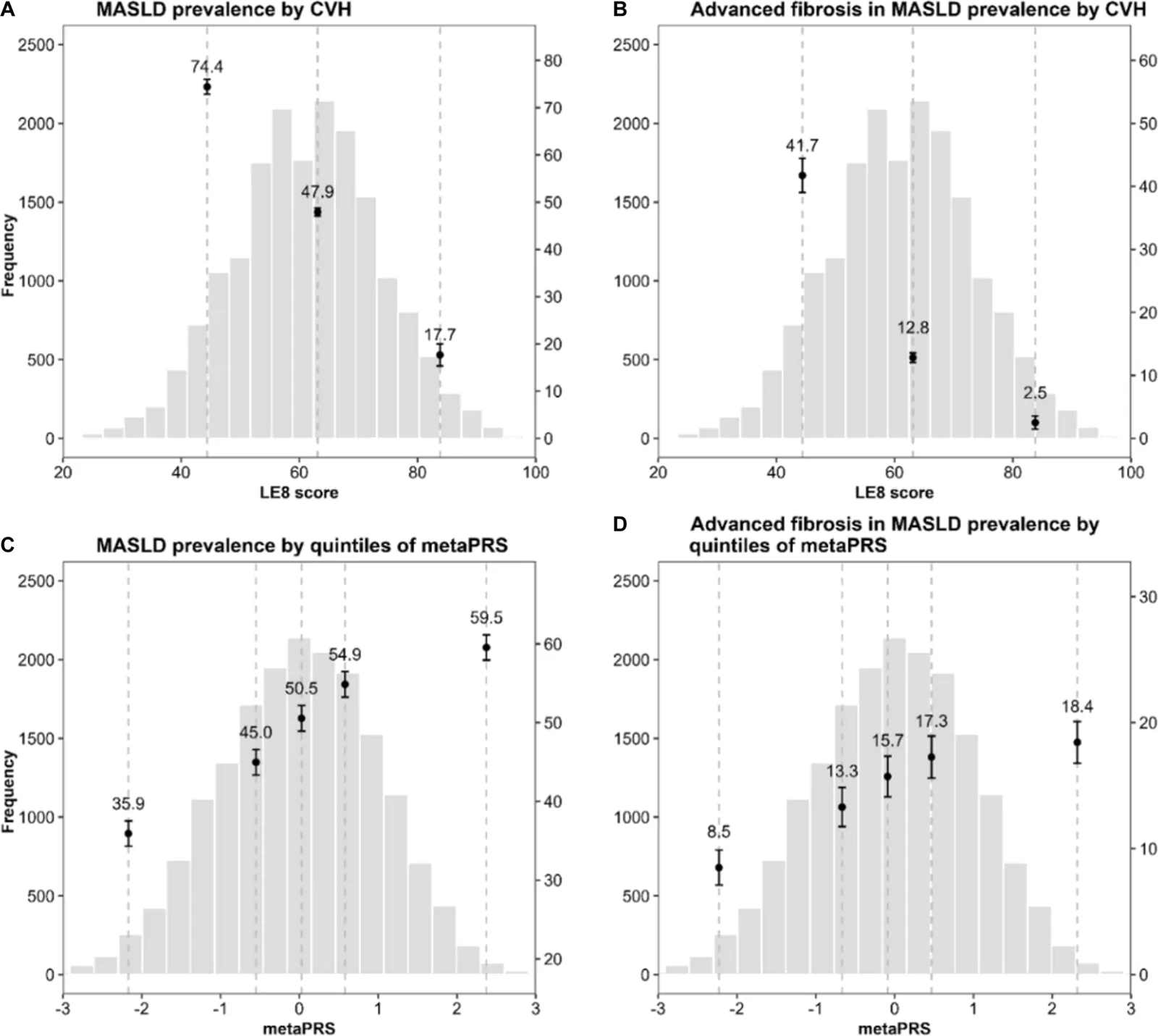
Prevalence of MASLD and advanced fibrosis in MASLD by CVH and quintiles of metaPRS. (A) Prevalence of MASLD by CVH and histogram of LE8 CVH score. (B) Prevalence of advanced fibrosis in MASLD by CVH and histogram of LE8 CVH score. (C) Prevalence of MASLD by quintiles of metaPRS and histogram of metaPRS. (D) Prevalence of advanced fibrosis in MASLD by quintiles of metaPRS and histogram of metaPRS. Gray dotted lines indicate metaPRS quintiles or 50th percentile of LE8 score by CVH, while dots and black lines indicate prevalence rates of MASLD or advanced fibrosis in MASLD and their 95% CIs. Prevalence rates and 95% CIs were standardized by age, sex, and region. CI, confidential interval; CVH, cardiovascular health; LE8, Life’s Essential 8; MASLD, metabolic dysfunction-associated steatotic liver; PRS, polygenetic risk score.

### Interactions and joint associations of CVH and metaPRS with MASLD and advanced fibrosis in MASLD

Interactions were observed between CVH (measured by LE8) and metaPRS on MASLD (*P* value for interaction < 0.02). The inverse association between LE8 and MASLD risk was stronger among those with a high genetic risk of MASLD compared with intermediate or low genetic risk (Fig. 4). ORs in descending genetic risk subgroups were 0.41 (0.37 to 0.45), 0.46 (0.43 to 0.48), and 0.47 (0.43 to 0.51), respectively (Table 2). Similar interactions were observed when the psychological health component was tentatively added into LE8 (*P* value for interaction by metaPRS < 0.03; Table S7).

**Fig. 4.**
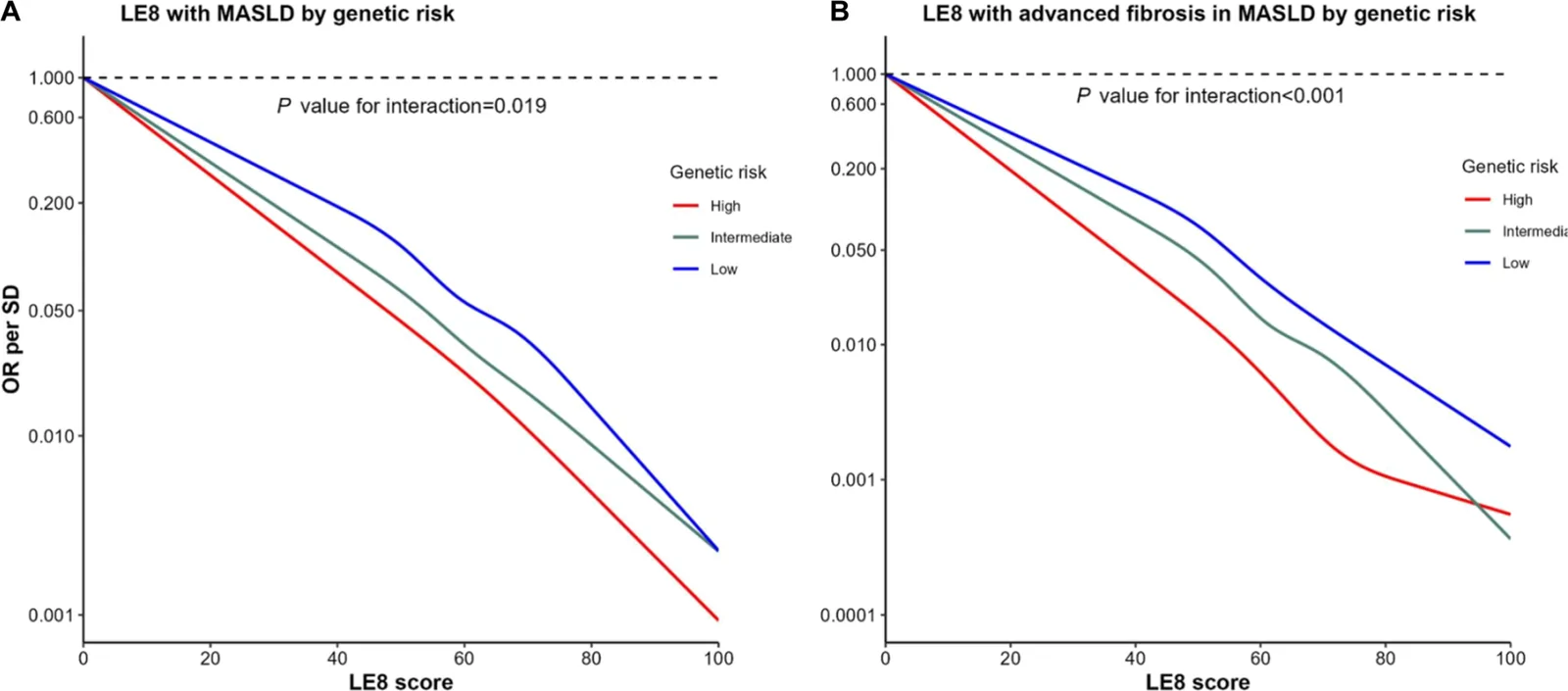
Dose–response relationships between LE8 scores and (A) MASLD and (B) advanced fibrosis in MASLD by genetic risk. Solid lines represent ORs by genetic risk. Restricted cubic spline regressions with 5 knots placed at the 5th, 25th, 50th, 75th, and 95th percentiles were used in each genetic risk group. The models were adjusted for age, sex, region, marital status, household income, and education. The genetic risk was categorized as high (top quintile), intermediate (2nd to 4th quintiles), and low (bottom quintile) according to quintile of metaPRS. PRS, polygenetic risk score; LE8, Life’s Essential 8; MASLD, metabolic dysfunction-associated steatotic liver disease.

When genetic risk and LE8 were combined, there was a monotonic association of the risk of MASLD with decreasing genetic risk and increasing CVH (Fig. 5). Compared with participants with a high genetic risk of MASLD and a low CVH profile, those with a low genetic risk of MASLD and a high CVH had a 98% lower risk of MASLD (OR = 0.02 [0.01 to 0.03]). Likewise, interactions and joint associations were identified between CVH (quantified by LE8 scores) and metaPRS on advanced fibrosis (≥F3, LSM ≥ 10.0 kPa) in MASLD (*P* value for interaction < 0.001) (Table 2 and Figs. 3 to 5).

**Fig. 5.**
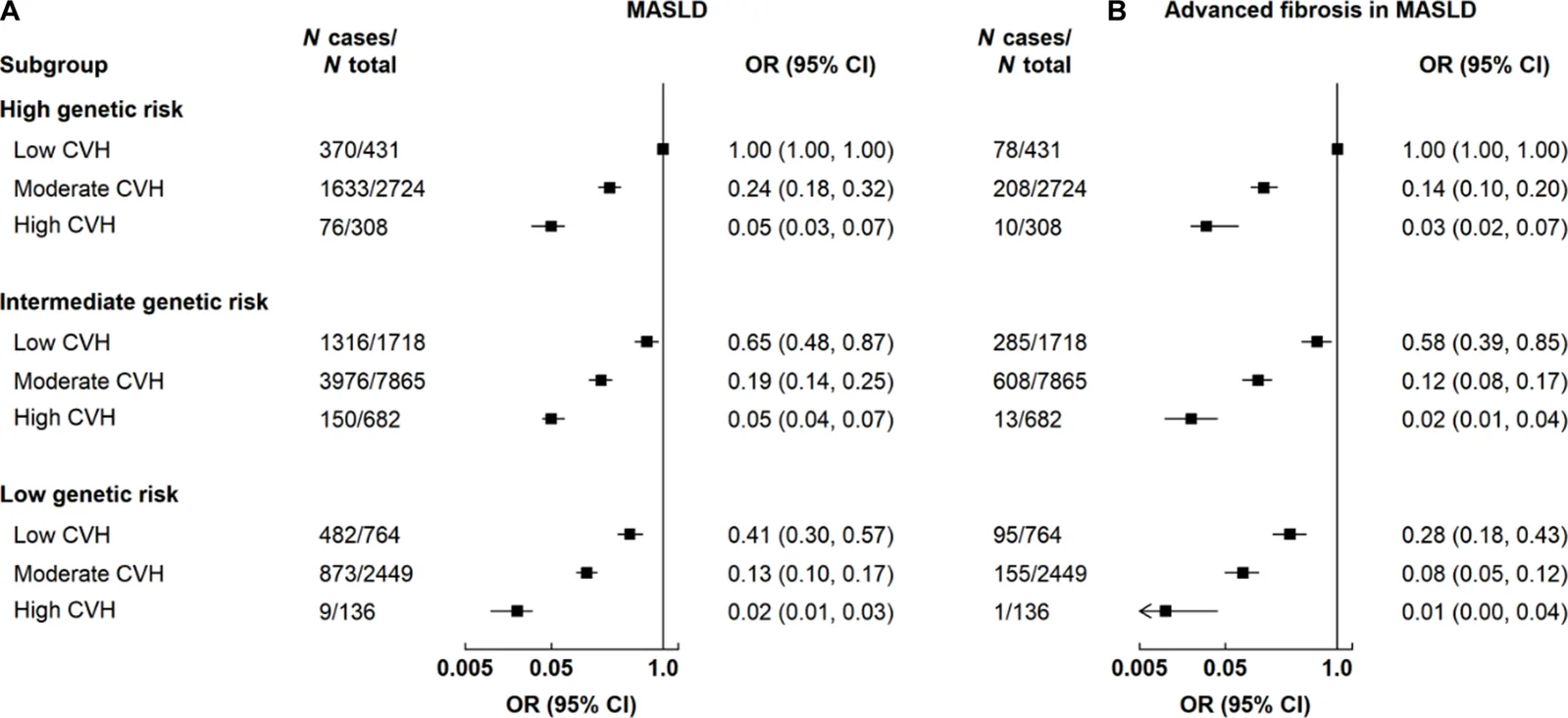
Joint associations of metaPRS and LE8 with MASLD and advanced fibrosis in MASLD. Boxes represent the ORs for (A) MASLD and (B) advanced fibrosis in MASLD associated with LE8 across strata of metaPRS. The multivariable logistic regression model was used in the analysis, adjusting for age, sex, region, marital status, household income, and education. CI, confidence interval; CVH, cardiovascular health; MASLD, metabolic dysfunction-associated steatotic liver disease; OR, odds ratio; PRS, polygenetic risk score.

### Sensitivity and subgroup analyses

Results remained consistent with the primary findings when genetic risk was categorized into low, intermediate, and high based on tertiles of the metaPRS (Table S9). Furthermore, the protective associations between CVH (measured by LE8) and MASLD were consistent across different degrees of liver steatosis and fibrosis, and interactions between LE8 and metaPRS were observed (*P* value for interaction < 0.001; Table S10). In subgroup analyses, the protective associations of LE8 score with MASLD were stronger among women and younger adults (*P* value for interaction < 0.05). Additionally, the protective associations were stronger with advanced fibrosis in MASLD among women (*P* value for interaction < 0.001; Table S11).

## Discussion

There were 3 primary findings of the current study. First, we showed that a superior CVH profile, defined by LE8, was associated with appreciably >90% lower risk of MASLD and advanced fibrosis in MASLD in Chinese adults. Second, we constructed a metaPRS for MASLD and demonstrated better risk stratification compared with NAFLD PRS. Third, the impact of LE8 on MASLD and advanced fibrosis risk was modified by metaPRS for MASLD. Individuals with a higher genetic risk of MASLD appeared to benefit more from a high LE8 score for both MASLD and advanced fibrosis among MASLD. Our findings emphasized the pivotal role of CVH and genetic risk in MASLD and advanced fibrosis in the Chinese population.

The CVH score among Chinese population in this study was comparable to that of National Health and Nutrition Examination Survey (NHANES) participants within a similar age range of 65 to 79 years (mean: 61.4 versus 62.3) [34]. Our findings suggest that achieving moderate and ideal CVH, as indicated by LE8, could result in ~70% to ~95% lower risks of MASLD and advanced fibrosis in MASLD, compared with those who had low CVH. These results aligned with previous studies conducted in North America, Europe, and East Asia, involving 1,812 to 266,645 adults, which reported that participants in the optimal CVH categories had 22% to 96% lower risk of fatty liver compared to those in the inadequate CVH categories (OR = 0.04 to 0.78) [29,34–36]. Consistent with these observational studies, mendelian randomization studies conducted in Europeans suggested that genetically determined smoking, BMI, T2D, systolic BP, HDL-C, and TG were associated with higher risk of NAFLD [37]. These findings suggested that lifestyle interventions targeting the components of LE8 have important implications in the prevention of MASLD and advanced fibrosis in MASLD. Recently, sufficient high-quality evidence has shown that psychological health characteristics (e.g., optimism, environmental mastery, and perceived reward from social roles) are associated with CVH and are foundational in achieving optimal and equitable CVH [6,7]. We tentatively included the psychological health component into LE8 to construct the LC9 [7] and found marked associations and interactions for LC9 compared with LE8. In summary, our study showed strong protective associations of CVH, which encompasses health behaviors, health factors, and health psychology, with MASLD and advanced fibrosis in MASLD, and this finding supported the broader application of CVH assessment for hepatic health promotion.

In our study, we employed the meta-analytic method to construct a meta-PRS by incorporating NAFLD and metabolic related trait-associated variants. The metaPRS method, which combines multiple risk alleles of diseases and related traits, has successfully demonstrated promise in risk stratification among the Chinese population, particularly in traditional diseases such as CAD and stroke [17,18]. This approach extends beyond traditional diseases and amalgamates information from various MASLD-related traits and provides a holistic and comprehensive assessment, highlighting its potential as a valuable tool for stratification. Like metaPRS for CVD, the metaPRS for MASLD exhibited better performance in risk stratification compared to NAFLD PRS or trait-specific PRS (Fig. S8). The performance of metaPRS in the current study was better than previous studies conducted in Europeans relying on NAFLD PRS and ascertaining NAFLD primarily by the International Classification of Diseases, Tenth Revision (ICD-10) code (Table S12). The majority of previous studies constructed unweighted PRS from common variants (e.g., PNPLA3 and TM6SF2), and it may be difficult to provide precise risk stratification for MASLD and advanced fibrosis in MASLD [38].

By utilizing metaPRS for MASLD, our study identified an interaction between an individual’s CVH status (measured by LE8) and genetic susceptibility in influencing the prevalence of MASLD and advanced fibrosis in MASLD. Stratified analyses revealed that the protective associations of LE8 with MASLD and advanced fibrosis in MASLD were more prominent among those with higher genetic susceptibility. These findings align with prior studies on gene–lifestyle interactions, highlighting the modifying effects of genetic risk on diet, BMI, and other CVH-related factors in influencing risk of liver steatosis and advanced fibrosis (Table S13). A multi-ethnic prospective study involving 101,828 participants demonstrated that adiposity amplified the effect of 3 variants (PNPLA3, TM6SF2, and GCKR) associated with NAFLD (*P* value for interaction: 5.5 × 10^−5^ to 0.006) [39]. Nevertheless, there is scarce evidence on the interactions of genetics with compositive CVH status on MASLD and advanced fibrosis in MASLD and results were inconsistent. One study from the Framingham Heart Study involving 2,773 participants showed that compared to those at lower NAFLD genetic risk, individuals with higher genetic risk experienced a higher relative decrease in liver fat accumulation with each CVH metric promotion (defined by Life’s Simple 7; *P* value for interaction: 0.04) [36]. Recently, another study from the UK Biobank involving 266,645 participants did not observe interaction between genetic risk of NAFLD and LE8 on new-onset severe MASLD (*P* value for interaction: 0.835; Table S14) [40]. Our study adds to the evidence base on the interaction between CVH and genetic risk in influencing the risk of MASLD and advanced fibrosis in MASLD.

The beneficial role of LE8 in MASLD reinforced the recommendations from the European Association for the Study of the Liver (EASL) and the American Association for the Study of Liver Diseases (AASLD), which emphasize the importance of lifestyle modifications and management of CVH risks in the prevention and management of liver steatosis and advanced fibrosis [39,41,42]. Therefore, health education emphasizing incremental interventions on behavioral and metabolic risk factors could be beneficial. To our knowledge, our study is the first to explore the interaction between lifestyle, as measured by the LE8, and MASLD genetic susceptibility in Chinese. The combined results from our study and previous studies suggest that a healthy lifestyle can offset the risks of MASLD and advanced fibrosis in MASLD conferred by genetic predisposition. However, longitudinal studies are warranted before personalized lifestyle recommendations can be made based on an individual’s genetic risk.

The strengths of this study included a substantial sample size from the large, nationally representative cohort of Chinese adults well-characterized for MASLD and CVH metrics, contributing to the robustness and generalizability of the findings. This study had several limitations. First, the assessment of lifestyle behavioral factors relied on self-reported data, which may introduce information bias. However, prior studies have demonstrated the high validity and reliability of CKB questionnaires (Supplementary Methods). For example, weighted kappa coefficients of major lifestyle behavioral factors ranged from 0.61 to 0.90. Moreover, any misclassification was likely nondifferential to MASLD status, which might bias the associations toward the null hypothesis [3]. Second, our study was constrained due to the unavailability of liver biopsy, which was considered the gold standard for diagnosing liver steatosis and fibrosis. However, the invasive nature of liver biopsy restricts its universal applicability. Meanwhile, FibroTouch is a valuable diagnostic tool that showed good diagnostic accuracy compared to biopsy [area under receiver operating curve (AUROC) 0.69 to 0.93 for steatosis, 0.71 to 0.93 for fibrosis] (Table S15). Meanwhile, FibroTouch is widely used in clinical settings as well as population-based surveys within China [27,43,44]. Furthermore, previous studies have shown a strong correlation between FibroTouch and FibroScan (commonly used in Western countries), with correlation coefficients ranging from 0.70 to 0.85 for LSM and 0.64 to 0.76 for FAP (Table S16) [43,45]. When compared to liver biopsy, FibroTouch exhibits diagnostic performance similar to FibroScan in grading steatosis and fibrosis (AUROC 0.69 to 0.93 versus 0.58 to 0.93 for steatosis, 0.71 to 0.93 versus 0.79 to 0.96 for fibrosis; Table S15) [43]. Additionally, we conducted sensitivity analyses using different cutoff values to confirm the main results (Table S10). Third, our study primarily enrolled participants with a mean age of 65.2 (range: 45.4 to 95.4), and the prevalence of liver steatosis was strikingly high. However, the prevalence of MASLD in our study was consistent with previous studies in China with similar age range (52.0% versus 31.5% to 51.6%; Table S17). Fourth, the examination of the associations between CVH, as assessed by LE8, and the risk of MASLD and advanced fibrosis are inherently cross-sectional, which precludes temporal inferences and may be subject to reverse causality. However, this concern is likely minimized in our study because MASLD is typically asymptomatic in its early stages [30]. Consequently, participants were less likely to have systematically modified the behavioral components of LE8, including diet, physical activity, smoking, and sleep, in response to their liver condition. Nonetheless, prospective cohort studies are warranted to confirm the temporal associations of these associations. Fifth, despite accounting for various confounding factors, the potential for unmeasured or residual confounding remained. Sixth, while elastic-net regression with 10-fold cross-validation was employed to minimize overfitting and enhance the internal stability of the constructed metaPRS for MASLD, the lack of independent cohort for external validation remains a limitation. Future studies are warranted to confirm the generalizability and predictive performance of this metaPRS in diverse populations.

## Conclusion

Our study in this Chinese population showed the importance of both CVH and genetic risk in the prevention of MASLD and advanced fibrosis in MASLD. Adhering to AHA LE8 metrics may provide greater benefits for those at higher genetic susceptibility for MASLD. These findings underscored the broader health implications of LE8 extending beyond CVH to include hepatic health promotion. The hepatology community should actively promote the incorporation of CVH metrics and genetic risk score stratification in the primary and secondary prevention of MASLD.

## Ethical Approval

The CKB complies with all the required ethical standards for medical research on human subjects. Ethical approvals were granted and have been maintained by the relevant institutional ethical research committees in the UK and China.

## Data Availability

Details of how to access China Kadoorie Biobank data and details of the data release schedule are available from https://www.ckbiobank.org/data-access.

## Appendix

Members of the China Kadoorie Biobank collaborative group are listed below.

**International Steering Committee:** Derrick Bennett, Junshi Chen, Zhengming Chen (PI), Rory Collins, Liming Li (PI), Jun Lv, Richard Peto, Dianjianyi Sun, Robin Walters, Canqing Yu.

**National Co-ordinating Centre, Beijing:** Liming Li, Jun Lv, Canqing Yu, Dianjianyi Sun, Yuanjie Pang, Yuting Han, Can Hou, Qingmei Xia, Chao Liu, Pei Pei, Lang Pan, Xiao Han, Honglu Bian.

**International Co-ordinating Centre, Oxford:** Daniel Avery, Maxim Barnard, Derrick Bennett, Ruth Boxall, Yiping Chen, Zhengming Chen, Jonathan Clarke; Robert Clarke, Huaidong Du, Ahmed Edris Mohamed, Hannah Fry, Prapthi Harish, Pek Kei Im, Andri Iona, Christiana Kartsonaki, Kshitij Kolhe, Hubert Lam, Kuang Lin, James Liu, Iona Millwood, Sam Morris, Qunhua Nie, Alfred Pozarickij, Maryam Rahmati, Paul Ryder, Maruf Sarder, Dan Schmidt, Becky Stevens, Iain Turnbull, Robin Walters, Baihan Wang, Lin Wang, Neil Wright, Ling Yang, Xiaoming Yang, Pang Yao.

**10 Regional Co-ordinating Centres:** Qingdao CDC: Zengchang Pang, Ruqin Gao, Kunzheng Lv, Shanpeng Li, Haiping Duan, Shaojie Wang, Yongmei Liu, Ranran Du, Liang Cheng, Xiaocao Tian, Hua Zhang. **Licang CDC:** Dan Hu, Xiaoyan Zheng, Yujie Wang. **Heilongjiang Provincial CDC:** Wei Sun, Shichun Yan, Yong Zhou. **Nangang CDC:** Chi Wang, Zhenyuan Wu, Lishun Zhai, Zhaoxi Pang, Shiwen Dong, Li Liu. **Hainan Provincial CDC:** Dapeng Yin, Bin He, Ying Liu, Xingren Wang, Tingting Ou. **Meilan CDC:** Xiangyang Zheng, Dewei Zheng, Shuai Yang, Lihui Li, Xingjiao Chen. **Jiangsu Provincial CDC:** Yan Xu, Jinyi Zhou, Ran Tao, Jian Su, Xikang Fan, Xuejia Chen, Yuxiao Huang. **Suzhou CDC:** Yan Lu, Yujie Hua, Li Xing, Shuxian Wang, Jianrong Jin, Juping Ma, Jinchao Liu, Kaifei Zhu, Hongfu Ren, Xingfeng Shen. **Guangxi Provincial CDC:** Ge Zhong, Wei Mao, Zhenzhen Lu, Yanxu Zhong. **Liuzhou CDC:** Lifang Zhou, Rong Pan, Jian Lan, Xiaoping Tan, Jinxue Tan, Yishan Xie, Liuping Wei, Liyuan Zhou, Sisi Wang. **Sichuan Provincial CDC:** Xianping Wu, Ningmei Zhang, Xiaofang Chen, Xiaoyu Chang, Zhuo Wang, Yujin He. **Pengzhou CDC:** Mingqiang Yuan, Ling Wang, Xiaofang Chen, Zhaodong Wang, Qiang Sun, Yang Lin. **Gansu Provincial CDC:** Faqing Chen, Xiaolan Ren, Lijun Chang, Feiming Zhong. **Maiji CDC:** Jianjun Feng, Weijie Hu, Xiaofang Zhang, Yalin Chen, Honghong Wang, Jun Wang. **Henan Provincial CDC:** Linqi Diao, Zhiwei Han, Dengjun Zhu, Kai Kang, Shixian Feng, Wenjie Yang, Huizi Tian, Yali Yan, Bing Han, Li Gao, Shaofang Li, Tianfang Xing, Wei Tang. **Huixian CDC:** Xiaolin Li, Huarong Sun, Xiaocong Zhao, Ying Li, Chen Hu, Pan He, Xukui Zhang, Yuanyuan Jin, Lan Luo. **Zhejiang Provincial CDC:** Min Yu, Ruying Hu, Hao Wang, Weiwei Gong, Jieming Zhong, Meng Wang, Chunxiao Xu, Keqing Gong. **Tongxiang CDC:** Hao Xu, Yuan Cao, Kaixu Xie, Lingli Chen, Xiaomei Tu, Junlong Pan. **Hunan Provincial CDC:** Xiaojun Li, Li Yin, Huilin Liu, Yuan Liu, Lei Yin, Xian Xie, Jing Wang. **Liuyang CDC:** Bo Xiao, Zongwei Deng, Yuan Peng, Libo Zhang, Chan Qu, Li Deng, Qili Jiang, Yanling Chen.
