# Supplementary material for: Life’s Essential 8, Genetic Susceptibility, and Metabolic Dysfunction-Associated Steatotic Liver Disease in Chinese Adults
Source: Health Data Sci. 2026 Oct 19;6:0416. doi: 10.34133/hds.0416 (PMC13678080; doi:10.34133/hds.0416)
Supplement: Supplementary file 1 [file hds.0416.f1.docx]

# On-line Supplementary Material

# Life's Essential 8, Genetic Susceptibility, and Metabolic Dysfunction-Associated Steatotic Liver Disease in Chinese

# Health Data Science

Shuyao Song, BM^1*^, Jiaqi Liu, BM^1*^, Ting Wu, BM^1^, Jun Lv, PhD^1,2,3^, Canqing Yu, PhD^1,2,3^, Dianjianyi Sun, PhD^1,2,3^, Lang Pan, MSc^1,2^, Pei Pei, MSc^1,2^, Ling Yang, PhD^4,5^, Yiping Chen, DPhil^4,5^, Huaidong Du, PhD^4,5^, Fiona Bragg, DPhil^4,5^, Iona Millwood, DPhil^4,5^, Robin Walters, PhD^4,5^, Xiaojun Li, BS^6^, Dan Schmidt, MS^5^, Maxim Barnard, MS^5^, Junshi Chen, MD^7^, Zhengming Chen, DPhil^5^, Liming Li, MD^1,2,3^, Yuanjie Pang, DPhil^1,3^ on behalf of the China Kadoorie Biobank Collaborative Group**

*Joint first authors

**The members of steering committee and collaborative group are listed in Supplementary material.

1. Department of Epidemiology & Biostatistics, School of Public Health, Peking University, Beijing 100191, China
2. Peking University Center for Public Health and Epidemic Preparedness & Response, Beijing 100191, China
3. Key Laboratory of Epidemiology of Major Diseases (Peking University), Ministry of Education
4. Medical Research Council Population Health Research Unit at the University of Oxford, Oxford, United Kingdom
5. Clinical Trial Service Unit & Epidemiological Studies Unit (CTSU), Nuffield Department of Population Health, University of Oxford, United Kingdom
6. Jili Community Health Service, Liuyang, Hunan 410300, China
7. China National Center for Food Safety Risk Assessment, Beijing 100022, China

**Address for correspondence:**

Dr. Yuanjie Pang

Dept. of Epidemiology and Biostatistics

School of Public Health

Peking University

38 Xueyuan Road

Beijing, 100191, China

Tel: 86-010-82801528

Fax: 86-010-82801530

yuanjie_p@163.com

**Word count:** abstract 276, main text 4319

(2 tables, 5 figures and 25 supplementary tables/figures)

April 2026

**Table of content**

[Members of the China Kadoorie Biobank collaborative group	3](#_Toc11216)

[Supplementary Methods	4](#_Toc8216)

[Supplementary Table 1. Basic characteristics of the 3rd resurvey participants stratified by with and without genotyping information	21](#_Toc28953)

[Supplementary Table 2. Overview of the sources of genome-wide association studies for NAFLD PRS	22](#_Toc10998)

[Supplementary Table 3. The sources of genome-wide association studies for the selected variants for each trait PRS	23](#_Toc4978)

[Supplementary Table 4. Genetic variants selected for the construction of the trait-specific PRS and metaPRS	24](#_Toc440)

[Supplementary Table 5. Basic characteristics according to MASLD and advanced fibrosis	26](#_Toc16869)

[Supplementary Table 6. Associations of Life’s Essential 8 with mortality	27](#_Toc6607)

[Supplementary Table 7. Associations of Life’s Crucial 9 with MASLD and advanced Fibrosis in MASLD and linear associations by stratum of genetic risk	2](#_Toc23663)8

[Supplementary Table 8. Associations between each trait PRS and metaPRS with MASLD and advanced fibrosis in MASLD	2](#_Toc14954)9

[Supplementary Table 9. Associations of LE8 with MASLD and advanced fibrosis in MASLD stratified by and jointly with genetic risk categories (based on metaPRS tertiles)	3](#_Toc24600)0

[Supplementary Table 10. Sensitivity analysis of associations between LE8 scores with MASLD and fibrosis in MASLD by stratum of metaPRS	31](#_Toc23708)

[Supplementary Table 11. Associations of LE8 with MASLD and advanced fibrosis in MASLD in subgroup analysis	32](#_Toc22804)

[Supplementary Table 12. The associations of genetic risk with NAFLD, advanced severe liver diseases in previous studies	33](#_Toc7571)

[Supplementary Table 13. Interaction analysis of genetic factors and CVH in previous studies on NAFLD and advanced severe liver diseases	34](#_Toc12947)

[Supplementary Table 14. Associations of CVH with NAFLD by stratum of metaPRS in previous studies	35](#_Toc23435)

[Supplementary Table 15. The comparison of diagnostic performance of FibroTouch and FibroScan with liver biopsy	36](#_Toc7017)

[Supplementary Table 16. The comparison of FibroTouch and FibroScan attenuation parameter and liver stiffness measurement	37](#_Toc7875)

[Supplementary Table 17. Comparison of the prevalence of MASLD in this study with previous comparable studies with mean or median age ranging from 40 to 65	3](#_Toc1059)8

[Supplementary Figure 1. Flow chart of the study	3](#_Toc7785)9

[Supplementary Figure 2. MetaPRS flow diagram	4](#_Toc26200)0

[Supplementary Figure 3. Correlation plot of NAFLD and related trait-specific PRS	41](#_Toc10358)

[Supplementary Figure 4. Distribution of overall Life's Essential 8 score and each component of Life's Essential 8	42](#_Toc14796)

[Supplementary Figure 5. Dose-response relationships between Life's Essential 8 scores and (A) MASLD and (B) advanced fibrosis in MASLD	43](#_Toc20073)

[Supplementary Figure 6. Association of each component of Life's Essential 8 metrics and psychological health with MASLD and advanced fibrosis in MASLD	4](#_Toc1957)4

[Supplementary Figure 7. Associations of trait-specific PRS and metaPRS with MASLD	45](#_Toc17389)

[Supplementary Figure 8. Prevalence of MASLD by quintiles of metaPRS and each traits-specific PRS	46](#_Toc12178)

## Members of the China Kadoorie Biobank collaborative group

**International Steering Committee:** Junshi Chen, Zhengming Chen (PI), Robert Clarke, Rory Collins, Yu Guo, Liming Li (PI), Jun Lv, Richard Peto, Robin Walters. **International Co-ordinating Centre, Oxford:** Daniel Avery, Derrick Bennett, Ruth Boxall, Sue Burgess, Ka Hung Chan, Yumei Chang, Yiping Chen, Zhengming Chen, Johnathan Clarke; Robert Clarke, Huaidong Du, Ahmed Edris Mohamed, Zammy Fairhurst-Hunter, Hannah Fry, Simon Gilbert, Alex Hacker, Mike Hill, Michael Holmes, Pek Kei Im, Andri Iona, Maria Kakkoura, Christiana Kartsonaki, Rene Kerosi, Kuang Lin, Mohsen Mazidi, Iona Millwood, Sam Morris, Qunhua Nie, Alfred Pozarickij, Paul Ryder, Saredo Said, Sam Sansome, Dan Schmidt, Paul Sherliker, Rajani Sohoni, Becky Stevens, Iain Turnbull, Robin Walters, Lin Wang, Neil Wright, Ling Yang, Xiaoming Yang, Pang Yao.

**National Co-ordinating Centre, Beijing:** Yu Guo, Xiao Han, Can Hou, Jun Lv, Pei Pei, Chao Liu, Canqing Yu, Qingmei Xia. **10 Regional Co-ordinating Centres: Qingdao CDC:** Zengchang Pang, Ruqin Gao, Shanpeng Li, Haiping Duan, Shaojie Wang, Yongmei Liu, Ranran Du, Yajing Zang, Liang Cheng, Xiaocao Tian, Hua Zhang, Yaoming Zhai, Feng Ning, Xiaohui Sun, Feifei Li. **Licang CDC:** Silu Lv, Junzheng Wang, Wei Hou. **Heilongjiang Provincial CDC:** Wei Sun, Shichun Yan, Xiaoming Cui. **Nangang CDC:** Chi Wang, Zhenyuan Wu, Yanjie Li, Quan Kang. **Hainan Provincial CDC:** Huiming Luo, Tingting Ou. **Meilan CDC:** Xiangyang Zheng, Zhendong Guo, Shukuan Wu, Yilei Li, Huimei Li. **Jiangsu Provincial CDC:** Ming Wu, Yonglin Zhou, Jinyi Zhou, Ran Tao, Jie Yang, Jian Su. **Suzhou CDC:** Fang Liu, Jun Zhang, Yihe Hu, Yan Lu, Liangcai Ma, Aiyu Tang, Shuo Zhang, Jianrong Jin, Jingchao Liu. **Guangxi Provincial CDC:** Mei Lin, Zhenzhen Lu. **Liuzhou CDC:** Lifang Zhou, Changping Xie, Jian Lan, Tingping Zhu, Yun Liu, Liuping Wei, Liyuan Zhou, Ningyu Chen, Yulu Qin, Sisi Wang. **Sichuan Provincial CDC:** Xianping Wu, Ningmei Zhang, Xiaofang Chen, Xiaoyu Chang. **Pengzhou CDC:** Mingqiang Yuan, Xia Wu, Xiaofang Chen, Wei Jiang, Jiaqiu Liu, Qiang Sun. **Gansu Provincial CDC:** Faqing Chen, Xiaolan Ren, Caixia Dong. **Maiji CDC:** Hui Zhang, Enke Mao, Xiaoping Wang, Tao Wang, Xi zhang. **Henan Provincial CDC:** Kai Kang, Shixian Feng, Huizi Tian, Lei Fan. **Huixian CDC:** XiaoLin Li, Huarong Sun, Pan He, Xukui Zhang. **Zhejiang Provincial CDC:** Min Yu, Ruying Hu, Hao Wang. **Tongxiang CDC**: Xiaoyi Zhang, Yuan Cao, Kaixu Xie, Lingli Chen, Dun Shen. **Hunan Provincial CDC:** Xiaojun Li, Donghui Jin, Li Yin, Huilin Liu, Zhongxi Fu. **Liuyang CDC:** Xin Xu, Hao Zhang, Jianwei Chen, Yuan Peng, Libo Zhang, Chan Qu.

## Supplementary Methods

### 1. Cardiovascular health assessment with Life’s Essential 8

At the CKB baseline survey and subsequent resurveys, data collection was performed in temporary assessment clinics specifically set up in the local communities for this study. An interviewer-administered electronic questionnaire was used to collect information on sociodemographic factors, food frequency questionnaire, frequency, duration, and strength of physical activity, nicotine exposure, sleep information, and medication use. The definitions and scoring for the component metrics of Life’s Essential 8 (LE8) have been provided in ***Appendix 1*** (page 5). Each of the eight LE8 components was scored on a scale of 0-100 points, and then the aggregate score was scaled from 0 to 100 points, calculated as the unweighted average of all 8 component metric scores. The behaviour subscale was based on behavioural metrics (diet, physical activity, nicotine exposure, sleep health, and body mass index), and the biological subscale was based on biological metrics (blood lipids, blood glucose, and blood pressure), and both were scaled from 0 to 100 points. Overall LE8 scores of 80 to 100 be considered high cardiovascular health (CVH); 50 to 79, moderate CVH; and 0 to 49 points, low CVH.

### Appendix 1. Definition of LE8 metrics and scoring algorithm

| **CVH metric** | **Method of measurement** | **Quantification of CVH metric** | |
| --- | --- | --- | --- |
| **Diet** | Derived from self-report daily intake of 12 major food groups using factor analysis with the principal component method | Points | Modern dietary pattern score |
|  |  | 100 | Quintile 1 (ideal diet) |
|  |  | 80 | Quintile 2 |
|  |  | 50 | Quintile 3 |
|  |  | 25 | Quintile 4 |
|  |  | 0 | Quintile 5 (least ideal diet) |
| **PA** | Derived from all activity (leisure-time, work-related, commuting, and housework), MET-hour/day | Points | All activity MET-hours/day |
|  |  | 100 | Quintile 5 (≥17.2 MET-hours/day) |
|  |  | 80 | Quintile 4 (6.11-17.2 MET-hours/day) |
|  |  | 50 | Quintile 3 (3.23-6.11 MET-hours/day) |
|  |  | 25 | Quintile 2 (1.2-3.23 MET-hours/day) |
|  |  | 0 | Quintile 1 (<1.2 MET-hours/day) |
| **Smoking** | Self-reported use of cigarettes | Points | Combustible tobacco use |
|  |  | 100 | Never smoker |
|  |  | 80 | Occasional smoker |
|  |  | 60 | Ex regular smoker |
|  |  | 0 | Current smoker or stopped smoking because illness |
| **Sleep health** | Healthy sleep pattern index score from multiple sleep behaviours (sleep duration: 7 or 8 hours/day, without insomnia disorder, without snoring) | Points | Healthy sleep pattern index |
|  |  | 100 | 4 |
|  |  | 50 | 3 |
|  |  | 25 | 2 |
|  |  | 0 | 1 |
| **BMI** | Body weight in kilograms divided by height in metres squared | Points | BMI (kg/m^2^) |
|  |  | 100 | <23 |
|  |  | 70 | 23-25 |
|  |  | 30 | 25-30 |
|  |  | 15 | 30-35 |
|  |  | 0 | ≥35 |
| **Blood lipids** | LDL cholesterol calculation by plasma total cholesterol, HDL cholesterol, and total triglyceride. If drug-treated level, LDL add 2.3 mmol/L* | Points | LDL cholesterol (mg/dL) |
|  |  | 100 | <130 |
|  |  | 60 | 130-159 |
|  |  | 40 | 160-189 |
|  |  | 20 | 190-219 |
|  |  | 0 | ≥220 |
| **Blood glucose** | Random plasma glucose | Points | RPG (mmol/L) |
|  |  | 100 | ≤5.5 mmol/L |
|  |  | 80 | 5.6-6.8 mmol/L |
|  |  | 60 | 6.8-7.8 mmol/L |
|  |  | 30 | 7.8-11.1 mmol/L |
|  |  | 0 | ≥11.1 mmol/L (reference) |
| **BP** | Blood pressure measured by trained technicians, using calibrated instruments with standard protocols If drug-treated level, SBP add 15 mm Hg**^†^** | Points | SBP and DBP (mm Hg) |
|  |  | 100 | <120/<80 (optimal) |
|  |  | 75 | 120-129/<80 (elevated) |
|  |  | 50 | 130-139 or 80-89 (stage 1 hypertension) |
|  |  | 25 | 140-159 or 90-99 |
|  |  | 0 | ≥160 |

Abbreviations: BMI, body mass index; BP, blood pressure; CVH, cardiovascular health; MASLD, metabolic dysfunction-associated steatotic liver disease; PA, physical activity; SBP, Systolic Blood Pressure; DBP, Diastolic Blood Pressure.

778 participants had more than one missing of LE8. Missing values were filled separately using mean values for each combination of sex, age groups (<60, 60-80, ≥80), and the 10 regional centres.

*Blood lipids drug-treated level score was calculated refer to 2023 China Guidelines for Lipid Management. LDL values were adjusted for reported use of blood lipids-lowering medication by adding 2.22 mmol/L to the LDL values^1^.

**^†^**The blood pressure drug-treated level score was calculated to refer to previous CKB-related research. SBP values were adjusted for the reported use of blood pressure-lowering medication by adding (15 mm Hg) to the SBP values^2,3^.

### 2. Physical measurements in CKB

Height, weight, waist circumference, and blood pressure were recorded by trained technicians using calibrated instruments with standard protocols. Standing height and body weight were measured by a height meter and a body fat meter (Tanita BC418MA, ‎TANITA; Japan), respectively. Body mass index was calculated as weight in kilograms divided by height in meters squared. Blood pressure and heart rate measurements were taken twice for each participant by a digital sphygmomanometer (UA-767 PBT-Ci, A&D Instruments; UK). A third measurement was performed if the difference between the first two SBP measurements were greater than 10 mmHg, and the latter two readings were recorded. All measurements were carried out under standard operating instructions. Previous literature can be referred to for details.^2^

### 3. Assessment of LE8 biological metrics in CKB

For each participant, a 10 mL blood sample was collected without fasting requirement but with the time since the last meal was recorded. Random plasma glucose (RPG) was tested on-site using SureStep Plus meters (Lifescan, Johnson & Johnson). The participants with RPG of 7.8-11.0 mmol/L and were not diagnosed with diabetes before were invited to retest their fasting plasma glucose (FPG) the next morning. For clinical biochemistry tests, 4 ml blood samples collected and were centrifuged in the field, kept refrigerated at 4°C, and tested within 24 hours. Total cholesterol, triglyceride, and high-density lipoprotein cholesterol were assayed using the BS-240 clinical chemical analyzer (Mindray, China). Low-density lipoprotein cholesterol (LDL-C) was estimated based on total cholesterol, high-density lipoprotein cholesterol (HDL-C) and triglycerides using the Friedewald formula:^4^

*In mg/dL: LDL-C*$=total cholesterol -$*HDL-C* $- (0.2 \times triglycerides)$

### 4. Physical activity assessment in CKB

The questions on physical activity have been previously reported and were adapted from validated questionnaires used in several previous studies in both high-income and Chinese populations (e.g., the European Prospective Investigation into Cancer (EPIC) and Nutrition and the Shanghai Women’s Health Study (SWHS)), with some additional modifications after a CKB pilot study^5–8^. In baseline and subsequent resurveys, participants were asked about the frequency, duration, and type (intensity) of physical activity in four domains (i.e., occupation, commuting, housework, and leisure-time exercise) during the past 12 months. The questionnaire for physical activity information in CKB surveys was shown in ***Appendix 2*** (page 8-9). To quantify the amount of physical activity, metabolic equivalents (METs) from the 2011 update of a major compendium of physical activities were used (***Appendix 3***, page 10).^9^ The MET value for a particular type of physical activity represents the ratio of the energy expended per kilogram of body weight per hour during that activity relative to that expended when sitting quietly. For generic activities (e.g. ball games), mean MET scores of specific activities under that genre were used. If an activity listed in the compendium is not widely performed in China (e.g. paddleball), it was excluded from the calculation. To avoid overrepresentation of specific activities that happen to have multiple entries, mean MET scores were weighted by the reciprocal of the number of entries representing the same activity. The number of hours spent per day participating in each activity was multiplied by the MET value for that activity, and the daily amount of total physical activity was obtained by summing the MET-hours/day for activities related to occupational and nonoccupational (ie, commuting, housework and non-sedentary leisure time) activities. Although no separate validation study has been conducted on the CKB questionnaire, the observed associations of PA with adiposity and CVD risk in CKB provide support for the validity of the PAQ.^5,10^ In addition, data from CKB showed good reliability of the PAQ. In CKB, following the completion of the baseline survey, a quality control (QC) survey was done involving 923 participants with repeated measurements within 1 year after the baseline survey. Results indicated that the reliability of exercise participation (kappa=0.60) and total PA (intraclass correlation coefficient [ICC]=0.72) was reasonable.^11^The reliability of PAQ in CKB is comparable to other studies, including EPIC and SWHS, that estimated their ICC for total PA to be 0.75 (EPIC men), 0.68 (EPIC women), and 0.70 (SWHS), respectively.^8,12^ In addition, the kappa statistic for exercise participation is comparable to that in SWHS (0.64).

### Appendix 2. Questionnaire for the physical activity in the survey of China Kadoorie Biobank

Section A: For non-farmers

1. In the past 12 months, how active were you at work?

🞎 Mainly sedentary (e.g. office worker)

🞎 Standing occupation (e.g. guard, shop assistant)

🞎 Manual work (e.g. plumber, carpenter)

🞎 Heavy manual work (e.g. miner, construction worker)

🞎 Retired, housewife/husband, unemployed, or disabled 🡪 *go to Question 12*

2. In a typical week, about how many hours did you usually work? ___ hrs

3. In the past 12 months, how did you usually get to and from work?

🞎 Mainly walk	ed	🞎 By bicycle

🞎 By motorbike	🞎 By bus/car/ferry/train

🞎 Mainly stayed at home, or work near home 🡪 *go to Question 12*

4. How much time did you spend each day on the journey to and from work? ___ mins

Section B: For farmers

5. In the past 12 months, did your farming work change seasonally?

🞎 No	🡪	*go to Question 7*

🞎 Yes

6. In the farming season in the last 12 months:

How many months did the farming season usually last? ___ mths

What types of farming work did it usually involve?

🞎 Manual	🞎 Semi-mechanized	🞎 Fully mechanized

How many hours did you usually work each day? ___ hrs

Of which, how many hours did you sweat or have a much faster heartbeat? ___ hrs

7. In a typical week (in non-farming seasons), how many hours did you usually work in the field? ___hrs

8. Apart from the agriculture work, did you have any other job?

🞎 No	🡪 *go to Question 11*

🞎 Yes

9. How active were you at work with the other job?

🞎 Mainly sedentary	🞎 Mainly general manual work

🞎 Mainly standing	🞎 Mainly heavy manual work

10. In a typical week, about how many hours did you spent at the other job? ___hrs

11. In a typical day how much time did you usually spend on the journey to and from work on foot or by bicycle? ___ mins

Section C: For both farmers and non-farmers

12. In the past 12 months, how often did you exercise in your leisure time?

🞎 Never or almost never

🡪 *go to Question 15*

🞎 1-3 times/month

🞎 1-2 times/week	🞎 3-5 times/week

🞎 Daily or almost every day

13. What is your main type of exercise? *(tick one box only)*

🞎 Taichi /Qigong /Leisure walking	🞎 Brisk walking /Gymnastics /Folk dancing

🞎 Jogging /Aerobic exercise	🞎 Swimming

🞎 ‘Ball’ games (including also billiards, bowling, golf, tennis, table tennis and badminton)

🞎 Other (eg. mountain climbing, home exercises, and rope jumping)

14. About how many hours per week did you spent on these exercises? ___ hrs

15. In the past 12 months, how often did you sweat or have a much faster heartbeat because of exercise?

🞎 Never or almost never

🡪 *go to Question 17*

🞎 <1 time / week

🞎 1-2 times/week

🞎 3-5 times/week

🞎 Daily or almost every day

16. About how many hours per week did you do such vigorous activities? ___hrs

17. In the past 12 months, about how many hours per week did you do housework? ___ hrs

18. In the past 12 months, during leisure time, about how many hours per week did you spend on sitting activities (e.g. watching TV or reading)? ___ hrs

19. How many hours do you typically sleep per 24h day (incl. naps)? ___ hrs

* Information can also be found at http://www.ckbiobank.org/about-the-study/study-design

### Appendix 3. Physical activity types, MET values, codes and intensity categories*

| **Activity type** | **Intensity** | **MET** | **Codes*** |
| --- | --- | --- | --- |
| Heavy manual work | Vigorous | 6.5 | 11477 |
| Manual work | Moderate | 4.5 | 11476 |
| Standing work | Moderate | 3.8 | Mean of 11610 and 11630 |
| Sedentary work | Low | 1.8 | Mean of 11580, 11585, and 11590 |
| Manual work in the farming season | Vigorous | 6.3 | Mean of 11145 and 11146 |
| Semi-mechanized work in the farming season | Moderate | 3.4 | Mean of 11146 and 11147 |
| Fully mechanized work in the farming season | Low | 2.4 | Mean of 11147 and 11170 |
| Work outside the farming season | Low | 2 | 11147 |
| Walking | Moderate | 4 | 17270 |
| Bicycle | Vigorous | 6.8 | 1011 |
| Motorbike | Moderate | 3.5 | 16030 |
| Private or public transportation (such as bus, car, underground, and ferry) | Low | 1.7 | Mean of 16010, 16015, and 16016 |
| Household activity | Low | 2.8 | Mean of 05030†, 05040†, 05035, 05055, 05070, 05090†, 05092†, 05184, 05197, and 05200 |
| Tai-Chi/qigong/leisure walking | Moderate | 3.3 | Mean of 15670 and 17160 |
| Jogging/aerobic exercise | Vigorous | 7.4 | Mean of 03015, 12020, and 12150 |
| Ball games | Moderate | 5.5 | Mean of 15020†, 15030†, 15055, 15080, 15090, 15255, 15605†, 15610†, 15652, 15660, 15675, 15710†, and 15711† |
| Brisk walking/gymnastics/folk dancing | Moderate | 4.2 | Mean of 03025, 15300, and 17200 |
| Swimming | Vigorous | 7.2 | Mean of 18230, 18240, and 18310 |
| Other exercise, e.g. mountain walking, home exercise and rope jumping | Moderate | 5.9 | Mean of 02010, 02064, 04001, 04100, 15110†, 15120†, 15200, 15240, 15310, 15425†, 15430†, 15537, 15550‡, 15551‡, 15552‡, 15580, 15590, 15730, 15732‡, 15733‡, 15734‡, and 19030 |

Abbreviations: MET: Metabolic equivalent of tasks.

*Based on the 2011 Compendium of Physical Activities: a second update of codes and MET values. Ainsworth BE, et al. Medicine and Science in Sports and Exercise, 2011;43(8):1575-1581.

† Assigned 1/2 weight in calculating the mean MET value because the connecting two items represent one type of activity.

‡ Assigned 1/3 weight in calculating the mean MET value because the connecting three items represent one type of activity.

Reference: Du H, Bennett D, Li L, et al. Physical activity and sedentary leisure time and their associations with BMI, waist circumference, and percentage body fat in 0.5 million adults: the China Kadoorie Biobank study. Am J Clin Nutr. 2013;97(3):487-496.

### 5. Smoking assessment in CKB

Data collected on smoking included frequency, amount, and type of tobacco smoked, the ages at which participants started smoking regularly and stopped smoking, and the main reason for smoking cessation. Smoking status was classified as never, occasional, former regular, or current regular smoker. Never smokers were defined as those who reported not smoking at baseline and had smoked <100 cigarettes in lifetime. Former regular smokers (i.e. ex-regular) were defined as those who had smoked a total of at least 100 cigarettes or equivalent but had quit smoking for at least six months before baseline by choice. Occasional smokers were defined as those who did not meet the criteria for never smokers or former smoker, and who had not stopped smoking completely for at least the six months before baseline. Current regular smokers were defined as those who reported having ever smoked one or more cigarettes (or their equivalent) daily for at least six months. Among current regular smokers who had stopped six or more months before recruitment, approximately half did so because of ill health, and they were still counted as current regular smokers in the main analyses. Among current and former regular smokers, information was collected on the types and amount of tobacco smoked when last smoking. For those identified as ex-regular smokers, additional information was sought regarding the duration since they last smoked regularly, including both years and months.^11^

### 6. Sleep health assessment in CKB

Sleep health was measured by the healthy sleep pattern index score for individuals according to sleep information from the questionnaire. The questionnaire for sleep information in CKB surveys was shown in ***Appendix 4*** (page 12). Healthy sleep pattern index scores were calculated from multiple sleep behaviors. Participants were given one point for each of the three sleep patterns: (sleep duration: 7 or 8 hours/day, without insomnia disorder, without snoring). The present study defined the following sleep patterns. (a) Short/ long habitual sleep duration: participants were asked, “How many hours do you typically sleep per day (including naps)?” Sleep duration could only be reported on an hourly basis. Short sleep duration was defined as 6 h/d or less, while long sleep duration was defined as 9 h or more , according to the American National Sleep Foundation.^13^ (b) Insomnia disorder: participants were asked for the insomnia symptoms for at least 3 days/week in the past month, which were having difficulties in initiating or maintaining sleep (DIMS), early morning awakening (EMA), daytime dysfunction (DDF), and having to take medicine (herbal or sleeping pills) at least once a week to help sleep. According to the Diagnostic and Statistical Manual of Mental disorders (version 4) (DSM-4), those who reported either DIMS or EMA and reported DDF, or those who reported taking medicines to help sleep, were classified as having insomnia disorder.^14^ (c) Snoring: participants self-reported their usual snoring status during sleep as often, sometimes, and never/I do not know.^15^ Those who chose the first two options were classified into the snoring group, and others were in the no snoring group. In sleep health assessment in CKB, sleep duration (Spearman correlation coefficient = 0.63) and snoring (weighted Kappa = 0.69) showed good reproducibility among 15,720 participants who completed a repeated questionnaire survey within 1~2 weeks after baseline.^15^

### Appendix 4. Questionnaire for the sleep patterns in the survey of China Kadoorie Biobank

1. How many hours do you typically sleep per day (including naps)? ________ hours.
2. During the past month, did you have any of the following for ≥3 days each week?
3. Taking > 30 minutes to fall asleep after going to bed or waking up in the middle of the night. (Yes/No)
4. Waking up early and not being able to go back to sleep. (Yes/No)
5. Needing to take medicine (including herbal or sleeping pills) at least once a week to help sleep. (Yes/No)
6. Having difficulty staying alert while at work, eating or meeting people during daytime. (Yes/No)
7. Do you usually take a daytime nap? (Yes, usually / Yes, but only in summer / No)
8. Do you snore during sleep? (Yes, usually / Yes, sometimes / No or don’t know)

### 7. Diet assessment in CKB

At the CKB survey, data collection was performed in temporary assessment clinics especially set up in the local communities for this study. In the 3rd resurvey, dietary data was assessed by a semi-qualitative food frequency questionnaire (FFQ) covering 19 food groups (***Appendix 5***, page 15): rice, wheat products, other staple foods, meat, poultry, fish, eggs, milk, yogurt, other dairy foods (milk powder, cheese, etc.), fresh vegetables, dried vegetables, salted vegetables, pickled vegetables, fresh fruit, soybean, soymilk, juice, and sugar drink. Respondents were asked about the frequency of habitual consumption during the previous 12 months and chose among five categories of frequency (daily, 4 to 6 days per week, 1 to 3 days per week, monthly, or never or rarely). In addition, participants were asked about the amount of consumption. The questions were “During the past 12 months, about how often did you eat (food)?” and “on days when you consume it, how much do you consume?” For each participant, 12 major food groups and sweetened beverages consumption per week were estimated according to previous CKB research.

Diet quality was evaluated by the modern dietary pattern score derived from the consumption of major food groups and sweetened beverages using factor analysis with the principal component method.^16^ The factors were rotated by orthogonal transformation (varimax rotation) to maintain independent factors and greater interpretability. The number of factors (dietary patterns) was determined based on eigenvalue (>1), scree plot, factor interpretability, and the variance explained by each factor. The second factor, labeled as “modern dietary pattern”, had high loadings of animal-origin foods, vegetables, and fruit. The dietary pattern scores for each participant were calculated by summing the consumption of each food group, which was weighted by its factor loading. The factor loading matrix of modern dietary pattern was shown in ***Appendix 6*** (page 16). A higher score indicated higher compliance with the corresponding dietary pattern. The score was divided into quartiles (Q1 to Q4 in ascending order) in urban and rural areas separately in subsequent analysis. The modern dietary pattern score has been shown associated with the risk of cardiometabolic diseases and obesity (including general obesity and central obesity) in previous CKB studies.^17,18^

Two surveys have been conducted to assess the reproducibility and validity of CKB FFQs, respectively.^19,20^ To assess the reproducibility of the responses, about 5% of randomly chosen surviving participants in 10 survey sites were resurveyed between August and October of 2008. To assess the validation of the FFQ, a total of 432 participants were recruited between September 2015 and August 2016 who completed 12-day and 24-hour dietary recalls with FFQ. Spearman's coefficient and weighted kappa indicated good reproducibility and validity of major dietary factors (weighted kappa coefficients ranged from 0.61 to 0.90; ***Appendix 7***, page 16).

### Appendix 5. Questionnaire for the FFQ in the survey of China Kadoorie Biobank

1. During the past 12 months, about how often did you consume the following foods or drinks?

### Appendix 6. Factor loading of the modern dietary pattern by factor analysis with a principal component

| **Food groups** | **Factor loading** |
| --- | --- |
| Rice | -0.06 |
| Wheat | 0.06 |
| Other staple foods | -0.17 |
| Meat | **0.48** |
| Poultry | **0.54** |
| Fish | **0.47** |
| Eggs | 0.35 |
| Fresh vegetables | 0.29 |
| Fruit | **0.59** |
| Dried vegetables | **0.56** |
| Preserved vegetables | 0.17 |
| Soybean | **0.48** |
| Dairy | 0.39 |
| Sugar sweet beverages | 0.19 |
| Variance explained (%) | 14.93 |

Figures in bold indicate absolute factor loadings were greater than 0.4

### Appendix 7. The relative validity and reproducibility of food frequency questionnaires in the CKB

|  | Relative validity | |  | Reproducibility | |
| --- | --- | --- | --- | --- | --- |
|  | (Compared with 12-day 24h DRs) | |  | (Compared two qualitative FFQs) | |
|  | Weighted Kappa | Adjusted Spearman |  | Weighted Kappa | Adjusted Spearman |
| Rice | 0.90 | 0.56 |  | 0.88 | 0.54 |
| Wheat products | 0.81 | 0.43 |  | 0.8 | 0.37 |
| Other staple foods | 0.85 | 0.28 |  | 0.8 | 0.23 |
| Meat | 0.77 | 0.36 |  | 0.61 | 0.34 |
| Poultry | 0.62 | 0.26 |  | 0.64 | 0.25 |
| Fish/seafood | 0.75 | 0.49 |  | 0.73 | 0.59 |
| Eggs | 0.77 | 0.41 |  | 0.65 | 0.49 |
| Fresh vegetables | -0.01 | -0.03 |  | 0.06 | 0.02 |
| Fresh fruits | 0.81 | 0.42 |  | 0.72 | 0.53 |
| Soya | 0.65 | 0.17 |  | 0.65 | 0.36 |
| Preserved vegetables | 0.75 | 0.31 |  | 0.81 | 0.39 |
| Dairy products | 0.82 | 0.39 |  | 0.75 | 0.47 |

Abbreviations: DR, dietary recalls; FFQ, food frequency questionnaire.

A validation study compared 12-day and 24-hour dietary recalls with FFQs designed by CKB to assess the relative validity, and two FFQs were administered to evaluate the reproducibility^19^. The weight for kappa was defined to be 1 if the frequency levels were in the same group, 0.5 if they were in adjacent groups, and 0 if they were in extreme groups. Spearman coefficients were adjusted for age, sex and region. Coefficients were not significant (*p*-value > .05). Most food items from the FFQ showed acceptable or even good relative validity and reproducibility. More than 95% of participants consumed fresh vegetables every day.

### 8. Psychological health in CKB and CVH assessment with Life’s Crucial 9

Psychological health was measured through metrics including social disconnectedness, perceived isolation, and the incidence of significant life events (i.e. victimization, traffic injuries, unemployment, or retirement). The questionnaire for psychological health in CKB surveys was shown in ***Appendix 8*** (page 18). Prior studies have established that indicators of social isolation and loneliness may heighten the vulnerability to mental disorders and pose various health risks.^21,22^ In efforts to enhance CVH, psychological well-being has been incorporated into the broader spectrum of cardiovascular care.

Over the past decades, key findings have illuminated that psychological health is one of the essential foundational context of maintaining or improving CVH.^23,24^ To evaluate integrated CVH that encompass psychological health, we tentatively expanded the Life's Essential 8 framework including psychological health components to the Life's Crucial 9, as endorsed by the American Heart Association.^25^ In this expansion, scores for social disconnectedness or perceived isolation, along with occurrences significantly impacting psychological health, were integrated into the assessment. The psychological health scoring algorithm in the Life's Crucial 9 CVH framework was presented in ***Appendix 9*** (page 18).

### Appendix 8. Questionnaire for psychological health measurement in this study in China Kadoorie Biobank

**1. Social interaction**

(1) How often do you feel that you lack companionship? (Often/Sometimes/Almost no)

(2) How often do you feel left out? (Often/Sometimes/Almost no)

(3) How often do you feel isolated from others? (Often/Sometimes/Almost no)

**2. Over the past two years have you had any of the following major events in your life?**

(1) Violence (Yes/No)

(2) Loss of job/retirement (Yes/No)

(3) Major injury or traffic accident (Yes/No)

### Appendix 9. Psychological health scoring algorithm in the Life's Crucial 9 CVH framework

| **CVH metric** | **Method of measurement** | **Quantification of CVH metric** | |
| --- | --- | --- | --- |
| Psychological health | Social disconnectedness or perceived isolation scores and occurrences with significant impact on psychological health* | Points | Modern dietary pattern score |
|  |  | 100 | disconnectedness or isolation scores ≤3 |
|  |  | 80 | disconnectedness or isolation scores=4  scores ≥4 or ≥ 1 significant impact incidence |
|  |  | 50 | disconnectedness or isolation scores≥5  or≥1 significant impact occurrence |
|  |  | 0 | disconnectedness or isolation scores≥5  and≥1 significant impact occurrence |

*Social disconnectedness or perceived isolation scores were calculated as the sum of scores reflecting the frequency of experiencing feelings of loneliness, being excluded by others, or being isolated from others, rated on a scale from 0 (almost never), 1 (sometimes) to 2 (often). Occurrences with significant impact on psychological health in past 2 years were included victimization, major injury or traffic accident, and unemployment or retirement.

### 9. Generation of the metaPRS

To construct a combined polygenetic risk score (metaPRS) for MASLD, eight genetic risk scores (PRS) for specific traits were separately constructed, including NAFLD and seven NAFLD related traits (BMI, TC, TG, HDL, LDL, BP, and T2D, **Supplementary Figure 2**). NAFLD PRS was developed using the genetic variants that showed genome-wide significant associations with NAFLD based on the previous GWASs (**Supplementary Table 2**), and NAFLD related PRS were developed according to previously constructed PRS in large-scale prospective Chinese cohort studies (**Supplementary Table 3**). The genetic variants that showed significant associations with MASLD (*p*-value <0.05) in this study were included in the construction of trait-specific PRS. NAFLD PRS was calculated by using 15 single-nucleotide polymorphisms (SNPs) extracted from published single genome-wide association and genome-wide meta-analysis studies.^26–30^ The effect allele was defined as the NAFLD risk-increasing allele in previous GWAS studies. BMI PRS was built using 62 SNPs reported by previous CKB studies.^29^ Briefly, BMI PRS was derived from sex-stratified GWAS of BMI in each of CKB and UKB, using rank inverse-normal transformed BMI (RINT BMI) derived separately for each sex. The effect allele was defined as the trait-increasing allele in the UKB sex-combined analysis. The PRS for TC, TG, HDL, LDL, BP, and T2D were built according to constructed PRS previously in large-scale prospective Chinese cohort studies (n= 41,271).^31,32^ The genetic variants that showed genome-wide significant associations (*P* < 5 × 10^−8^) with related traits were used. The effect allele was defined as the trait-increasing allele in previous GWAS studies. Eight trait-specific PRS were separately constructed by summing the number of corresponding risk alleles (0, 1, or 2) for each individual, weighted by the corresponding effect size of variants. The information on the variants and their weights used for each trait-specific PRS construction was provided in **Supplementary Table 4**. Each trait-specific PRS was standardized by calculating the z-score (zero mean, unit standard deviation), which were correlated with each other to different degrees (**Supplementary Figure 3**).

A combined PRS (metaPRS) was generated by integrating the eight trait-specific PRS and using the elastic-net logistic regression with a 10-fold cross-validation method in the R package 'glmnet'. This method was used widely in large-scale prospective studies of both Chinese and Western populations to construct metaPRS and improved genetic risk stratification for major diseases (e.g. stroke and coronary artery disease).^31–33^ The associations of the eight PRS with MASLD were assessed by adjusting for age, sex, and region. The model with the best performance, defined as the maximum cross-validated area under the receiver operator curve, was selected as the final metaPRS model. The metaPRS for MASLD was constructed by summing the variants from each trait-specific PRS weighted by adjusted estimates derived from the final model. The weights of all variants for metaPRS were provided in **Supplementary Table 4**. We further categorized metaPRS into low (lowest quintile), intermediate (quintiles 2-4), and high (highest quintile) genetic risk.

## Supplementary Table 1. Basic characteristics of the 3rd resurvey participants stratified by with and without genotyping information

| **Variable *** | **With genotyping Information** | **Without genotyping Information** |
| --- | --- | --- |
|  | **(n=17,972)** | **(n=7106)** |
| Age (SD), year | 65.5 (9.2) | 65.3 (8.9) |
| Female, % | 11397 (64.0) | 4702 (64.7) |
| **Socioeconomic and lifestyle factors** |  |  |
| ≥9 years of education, % | 2766 (17.9) | 1794 (18.8) |
| Household income ≥35,000 RMB/year, % | 13189 (74.2) | 5486 (75.2) |
| Ever regular smoking, % |  |  |
| Male | 4217 (64.2) | 1558 (66.6) |
| Female | 226 (2.4) | 90 (2.3) |
| Weekly drinking, % |  |  |
| Male | 1890 (29.1) | 699 (29.1) |
| Female | 224 (2.1) | 96 (1.8) |
| Total physical activity (SD), MET-h/day | 15.2 (13.9) | 15.3 (12.2) |
| Snoring, % | 9176 (51.2) | 3569 (51.1) |
| **Blood pressure and anthropometry** |  |  |
| SBP (SD), mmHg | 132.9 (21.1) | 133.7 (21.1) |
| DBP (SD), mm Hg | 77.9 (10.7) | 78.7 (10.5) |
| RPG (SD), mg/dL | 6.5 (2.6) | 6.5 (2.6) |
| LDL (SD), mg/dL | 3.3 (1.4) | 3.3 (1.3) |
| Waist circumference (SD), cm | 86.1 (9.7) | 85.7 (9.7) |
| BMI (SD), kg/m^2^ | 24.5 (3.5) | 24.4 (3.5) |
| **Liver VCTE measurement** |  |  |
| FAP (IQR), dB/m | 248.0 (52.0) | 251.0 (52.0) |
| LSM (IQR), kPa | 6.9 (3.0) | 7.0 (3.2) |
| Advanced fibrosis, % ^†^ | 2318 (16.3) | 1075 (17.8) |
| **Cardiovascular health** |  |  |
| LE8 score (SD) | 61.0 (12.2) | 60.8 (12.1) |
| LE8 score behavior scale (SD) | 57.1 (16.8) | 57.5 (15.9) |
| LE8 score biological scale (SD) | 77.6 (19.4) | 76.8 (19.6) |
| **Prior disease history, %** |  |  |
| Diabetes | 2559 (14.7) | 1198 (15.7) |
| Ischemic heart disease | 1777 (10.6) | 775 (9.4) |
| Stroke or TIA | 1164 (6.7) | 502 (6.7) |
| Hypertension | 6437 (35.5) | 2463 (36.4) |
| Family history of diabetes | 2469 (14.6) | 1233 (15.3) |
| Family history of cancer | 3985 (22.5) | 1673 (23.4) |
| **Medication use, %** |  |  |
| Statin medication | 899 (5.5) | 479 (5.6) |
| Antihypertensive | 5881 (32.5) | 2268 (33.3) |
| Diabetes treatment | 1932 (11.2) | 922 (12.0) |

Abbreviations: BMI, body mass index; VMET, metabolic equivalent of the task; SBP, systolic blood pressure; TIA, transient ischemic attack. Results were standardized by age, sex, and region where appropriate. Values are means (standard deviation, SD), median (interquartile range, IQR), or %.

## Supplementary Table 2. Overview of the sources of genome-wide association studies for NAFLD PRS

| **GWAS reference**  **(Pubmed ID)** | **Phenotype** | **Sample size** | **Ethnicity** |
| --- | --- | --- | --- |
| 35654975 | Imaging‐based NAFLD (CT/MRI)  (Z‐scores) | 44,289 | European‐American, African‐American, and  Hispanic American |
|  | NAFLD‐confirmed biopsy  (yes/no) | 7,397 cases and 56,785 controls | European‐American and Hispanic American |
| 36280732 | Imaging‐based NAFLD (MRI-derived PDFF) (Z‐scores) | 36,166 individuals | European |
|  | NAFLD by ICD-10 | 9,491 cases and 876,210 controls | European |
| 34841290 | NAFLD by ICD-10 | 8,434 cases and 770,180 controls | European |
| 32298765 | NAFLD by histology | 1,483 cases and 17,781 controls | European |
| 35124268 | NAFLD by ICD-10 | 3,711 cases/426,252 controls | European |
| 35023183 | Liver fat content | 5,581 individuals | Chinese |

Abbreviation: NAFLD, non-alcohol fatty liver disease.

## Supplementary Table 3. The sources of genome-wide association studies for the selected variants for each trait PRS

| **Traits** | **N. variants *** | | **Variant reference**  **(Pubmed ID)** | **Variants reference ethnicity** | **Weight Reference**  **(Pubmed ID or DOI)** | **Weight calculation method** | **Weight ethnicity** |
| --- | --- | --- | --- | --- | --- | --- | --- |
|  | **Reference study** | **PRS** |  |  |  |  |  |
| **TC** | 100 | 5 | 26582766 | East Asian | 29403010 | Single GWAS^†^ | East Asian |
| **TG** | 164 | 12 | 29083407 | East Asian | 29083407 | Single GWAS and meta-analysis | Trans-ethnic |
| **HDL** | 354 | 26 | 28334899 | Trans-ethnic | 28334899 | Genome-wide meta-analysis | Trans-ethnic |
| **LDL** | 82 | 4 |  |  |  |  |  |
| **BP** | 82 | 6 | 25249183 | Chinese | 29403010 | Single GWAS | East Asian |
|  |  |  | 26390057 | Trans-ethnic |  |  |  |
| **T2D** | 297 | 20 | 26818947 | East Asian | 30718926; 22158537 | Genome-wide meta-analysis | East Asian |
| **BMI** | 730 | 62 | 37869961 | Chinese | 10.21203/rs.3.rs-1832470/v1 | Genome-wide meta-analysis | European |
| **NAFLD** | 66 | 15 | 36280732 | European | 36280732 | Single GWAS | European |
|  |  |  | 35654975 | Trans-ethnic | 35654975 | Single GWAS | Trans-ethnic |
|  |  |  | 34841290 | European | 34841290 | Single GWAS | European |
|  |  |  | 32298765 | European | 32298765 | Genome-wide meta-analysis | European |
|  |  |  | 35124268 | European | 35124268 | Single GWAS | European |
|  |  |  | 35023183 | Chinese | 35023183 | Genome-wide meta-analysis | Chinese |

Abbreviation: BP, blood pressure; BMI, body mass index; GWAS, Genome-wide association studies; HDL, high-density lipoprotein cholesterol; LDL, low-density lipoprotein cholesterol; PRS, polygenetic risk score; T2D, type 2 diabetes; TC, total cholesterol; TG, triglycerides.

* NAFLD PRS was based on variants associated with NAFLD in previous large-scale GWAS, and the PRS for BMI, TC, TG, HDL, LDL, BP and T2D were according to genotyped variants from previously constructed PRS developed in prospective Chinese cohort studies^31^. Then, variants associated with MASLD in this study were chosen to construct PRS for each trait (threshold *p*-value was 0.05).

†Priority was given to studies including Chinese participants for the calculation of weights for lipid PRS (TC, TG, HDL and LDL PRS).

## Supplementary Table 4. Genetic variants selected for the construction of the trait-specific PRS and metaPRS

| **Genotyped variants** | **CHR: Pos_hg19** | **EA** | **Traits PRS** | | **MetaPRS weight** | **Genotyped variants** | **CHR: Pos_hg19** | **EA** | **Traits PRS** | | **MetaPRS weight** |
| --- | --- | --- | --- | --- | --- | --- | --- | --- | --- | --- | --- |
|  |  |  | **Trait** | **Weight** |  |  |  |  | **Trait** | **Weight** |  |
| rs28482847 | 1: 46595077 | T | BMI | 0.012 | 0.024 | rs12597579 | 16: 20257867 | T | HDL | 0.018 | 0.007 |
| rs12094437 | 1: 91185495 | C | BMI | 0.017 | 0.034 | rs1421085 | 16: 53800954 | T | HDL | 0.012 | 0.148 |
| rs71495049 | 10: 34014435 | A | BMI | 0.024 | 0.048 | rs663129 | 18: 57838401 | G | HDL | 0.021 | 0.009 |
| rs10824218 | 10: 76421216 | A | BMI | 0.013 | 0.025 | rs58542926 | 19: 19379549 | T | HDL | 0.024 | 0.021 |
| rs11001963 | 10: 78760959 | T | BMI | 0.012 | 0.023 | rs4420638 | 19: 45422946 | A | HDL | 0.094 | 0.000 |
| rs72838588 | 10: 102420171 | A | BMI | 0.015 | 0.029 | rs12463617 | 2: 629244 | A | HDL | 0.015 | 0.011 |
| rs1048932 | 11: 115044850 | C | BMI | 0.016 | 0.031 | rs11125936 | 2: 62871225 | C | HDL | 0.009 | 0.002 |
| rs1433980 | 11: 130870956 | C | BMI | 0.017 | 0.033 | rs35419456 | 2: 43725965 | C | HDL | 0.020 | 0.000 |
| rs11223204 | 11: 132652554 | G | BMI | 0.014 | 0.028 | rs6093446 | 20: 39780932 | A | HDL | 0.006 | 0.003 |
| rs7313220 | 12: 17196832 | A | BMI | 0.011 | 0.022 | rs738409 | 22: 44324727 | C | HDL | 0.010 | 0.022 |
| rs7132908 | 12: 50263148 | A | BMI | 0.030 | 0.059 | rs17843797 | 3: 124453022 | T | HDL | 0.012 | 0.002 |
| rs9579775 | 13: 20616557 | C | BMI | 0.021 | 0.041 | rs10513801 | 3: 185822353 | T | HDL | 0.093 | 0.009 |
| rs4477562 | 13: 54104968 | T | BMI | 0.031 | 0.061 | rs9815354 | 3: 41912651 | G | HDL | 0.015 | 0.000 |
| rs11628702 | 14: 21932592 | T | BMI | 0.009 | 0.018 | rs12897 | 3: 172115902 | A | HDL | 0.014 | 0.000 |
| rs8011566 | 14: 42939471 | A | BMI | 0.013 | 0.025 | rs17087335 | 4: 57838583 | G | HDL | 0.013 | 0.000 |
| rs7161194 | 14: 101529005 | A | BMI | 0.020 | 0.039 | rs261967 | 5: 95850250 | A | HDL | 0.014 | 0.005 |
| rs1947043 | 15: 53153950 | G | BMI | 0.012 | 0.024 | rs888789 | 5: 74988369 | G | HDL | 0.011 | 0.000 |
| rs8029980 | 15: 98260155 | T | BMI | 0.011 | 0.022 | rs832552 | 5: 56113850 | T | HDL | 0.016 | 0.000 |
| rs1421085 | 16: 53800954 | C | BMI | 0.075 | 0.148 | rs148910227 | 7: 80302116 | T | HDL | 0.342 | 0.000 |
| rs3751837 | 16: 3583173 | T | BMI | 0.015 | 0.030 | rs174546 | 11: 61569830 | C | LDL | 0.028 | 0.004 |
| rs10083803 | 16: 6701400 | C | BMI | 0.014 | 0.027 | rs10401969 | 19: 19407718 | T | LDL | 0.034 | 0.000 |
| rs2547033 | 16: 24743657 | G | BMI | 0.016 | 0.032 | rs4420638 | 19: 45422946 | G | LDL | 0.167 | 0.000 |
| rs9299 | 17: 46669430 | T | BMI | 0.012 | 0.027 | rs11125936 | 2: 62871225 | T | LDL | 0.052 | 0.002 |
| rs4516268 | 17: 1846831 | C | BMI | 0.025 | 0.048 | rs12077210 | 1: 65894160 | T | NAFLD | 0.395 | 0.019 |
| rs10438964 | 18: 42950629 | C | BMI | 0.015 | 0.030 | rs1337101 | 1: 219726100 | G | NAFLD | 0.052 | 0.003 |
| rs429358 | 19: 45411941 | T | BMI | 0.026 | 0.061 | rs56094641 | 16: 53806453 | G | NAFLD | 0.042 | 0.002 |
| rs3957285 | 19: 1891992 | A | BMI | 0.016 | 0.031 | rs9922619 | 16: 53831771 | T | NAFLD | 0.077 | 0.004 |
| rs350832 | 19: 4069426 | A | BMI | 0.020 | 0.040 | rs58542926 | 19: 19379549 | T | NAFLD | 0.329 | 0.021 |
| rs11881883 | 19: 46221726 | G | BMI | 0.002 | 0.004 | rs8107974 | 19: 19388500 | T | NAFLD | 0.490 | 0.024 |
| rs28394227 | 19: 49620521 | C | BMI | 0.014 | 0.027 | rs73001065 | 19: 19460541 | C | NAFLD | 0.281 | 0.014 |
| rs6751993 | 2: 635864 | G | BMI | 0.057 | 0.112 | rs17216588 | 19: 19664077 | T | NAFLD | 0.477 | 0.023 |
| rs13002767 | 2: 60285450 | A | BMI | 0.013 | 0.026 | rs10500212 | 19: 19723215 | T | NAFLD | 0.438 | 0.021 |
| rs2861685 | 2: 67837553 | T | BMI | 0.018 | 0.035 | rs429358 | 19: 45411941 | T | NAFLD | 0.211 | 0.061 |
| rs114964326 | 2: 146015229 | G | BMI | 0.035 | 0.070 | rs738409 | 22: 44324727 | G | NAFLD | 0.385 | 0.022 |
| rs12692738 | 2: 165558252 | C | BMI | 0.014 | 0.028 | rs738408 | 22: 44324730 | T | NAFLD | 0.267 | 0.013 |
| rs51195 | 2: 166953619 | C | BMI | 0.012 | 0.024 | rs3747207 | 22: 44324855 | A | NAFLD | 0.289 | 0.014 |
| rs9630985 | 2: 181607676 | C | BMI | 0.017 | 0.033 | rs17598226 | 4: 100496891 | C | NAFLD | 0.042 | 0.002 |
| rs56360953 | 2: 208158452 | T | BMI | 0.014 | 0.027 | rs7029757 | 9: 132566666 | G | NAFLD | 0.083 | 0.004 |
| rs4625852 | 2: 236762851 | G | BMI | 0.016 | 0.031 | rs11205760 | 1: 51174330 | T | T2D | 0.066 | 0.007 |
| rs117435593 | 20: 36562529 | G | BMI | 0.029 | 0.057 | rs2820443 | 1: 219753509 | T | T2D | 0.025 | 0.003 |

**Supplementary Table 4. continued**

| **Genotyped variants** | **CHR: Pos_hg19** | **EA** | **Traits PRS** | | **MetaPRS weight** | **Genotyped variants** | **CHR: Pos_hg19** | **EA** | **Traits PRS** | | | **MetaPRS weight** |
| --- | --- | --- | --- | --- | --- | --- | --- | --- | --- | --- | --- | --- |
|  |  |  | **Trait** | **Weight** |  |  |  |  | **Traits PRS** | | **Traits PRS** |  |
| rs2425841 | 20: 44905317 | A | BMI | 0.012 | 0.023 | rs11257655 | 10: 12307894 | T | T2D | 0.104 | | 0.011 |
| rs202076222 | 3: 42324697 | C | BMI | 0.023 | 0.045 | rs4757391 | 11: 16302939 | T | T2D | 0.018 | | 0.002 |
| rs148535477 | 3: 61264543 | T | BMI | 0.023 | 0.045 | rs174546 | 11: 61569830 | C | T2D | 0.035 | | 0.004 |
| rs55853915 | 3: 66487652 | G | BMI | 0.012 | 0.023 | rs9568867 | 13: 54107352 | A | T2D | 0.033 | | 0.004 |
| rs2016469 | 3: 108023965 | A | BMI | 0.013 | 0.026 | rs12597579 | 16: 20257867 | C | T2D | 0.068 | | 0.007 |
| rs1870931 | 3: 119757013 | C | BMI | 0.014 | 0.027 | rs1558902 | 16: 53803574 | A | T2D | 0.127 | | 0.014 |
| rs61791109 | 3: 170734377 | C | BMI | 0.018 | 0.035 | rs9299 | 17: 46669430 | T | T2D | 0.031 | | 0.027 |
| rs869400 | 3: 185826740 | G | BMI | 0.030 | 0.058 | rs663129 | 18: 57838401 | A | T2D | 0.083 | | 0.009 |
| rs6843852 | 4: 162132758 | T | BMI | 0.013 | 0.025 | rs58542926 | 19: 19379549 | T | T2D | 0.051 | | 0.021 |
| rs2307111 | 5: 75003678 | T | BMI | 0.029 | 0.057 | rs769449 | 19: 45410002 | G | T2D | 0.036 | | 0.004 |
| rs512921 | 5: 43134510 | G | BMI | 0.015 | 0.030 | rs12463617 | 2: 629244 | C | T2D | 0.097 | | 0.011 |
| rs1459843 | 5: 95867223 | C | BMI | 0.015 | 0.029 | rs11125936 | 2: 62871225 | C | T2D | 0.022 | | 0.002 |
| rs2233598 | 5: 140868499 | G | BMI | 0.013 | 0.026 | rs6093446 | 20: 39780932 | A | T2D | 0.030 | | 0.003 |
| rs11134999 | 5: 176163794 | C | BMI | 0.011 | 0.021 | rs738409 | 22: 44324727 | G | T2D | 0.029 | | 0.022 |
| rs796915 | 6: 154304628 | G | BMI | 0.014 | 0.027 | rs17843797 | 3: 124453022 | T | T2D | 0.019 | | 0.002 |
| rs10269783 | 7: 49616203 | A | BMI | 0.014 | 0.027 | rs10512861 | 3: 132257961 | G | T2D | 0.012 | | 0.001 |
| rs2404324 | 7: 99023461 | A | BMI | 0.023 | 0.045 | rs10513801 | 3: 185822353 | T | T2D | 0.084 | | 0.009 |
| rs12375196 | 7: 103416541 | A | BMI | 0.019 | 0.036 | rs261967 | 5: 95850250 | C | T2D | 0.043 | | 0.005 |
| rs4877313 | 9: 88915566 | A | BMI | 0.015 | 0.029 | rs174547 | 11: 61570783 | T | TC | 0.032 | | 0.000 |
| rs139887373 | 9: 122654933 | A | BMI | 0.019 | 0.038 | rs10401969 | 19: 19407718 | T | TC | 0.060 | | 0.000 |
| rs1019240 | 9: 122957315 | T | BMI | 0.011 | 0.022 | rs4420638 | 19: 45422946 | G | TC | 0.110 | | 0.000 |
| rs6597653 | 9: 133788465 | C | BMI | 0.013 | 0.026 | rs11125936 | 2: 62871225 | T | TC | 0.054 | | 0.002 |
| rs174546 | 11: 61569830 | T | BP | 0.013 | 0.004 | rs738409 | 22: 44324727 | C | TC | 0.021 | | 0.022 |
| rs4336994 | 11: 16302494 | G | BP | 0.017 | 0.000 | rs2820443 | 1: 219753509 | T | TG | 0.014 | | 0.003 |
| rs9568867 | 13: 54107352 | A | BP | 0.009 | 0.004 | rs11257655 | 10: 12307894 | T | TG | 0.025 | | 0.011 |
| rs1558902 | 16: 53803574 | A | BP | 0.009 | 0.014 | rs174546 | 11: 61569830 | T | TG | 0.033 | | 0.004 |
| rs9815354 | 3: 41912651 | A | BP | 0.008 | 0.000 | rs9568867 | 13: 54107352 | G | TG | 0.032 | | 0.004 |
| rs17843768 | 3: 124448385 | A | BP | 0.022 | 0.000 | rs663129 | 18: 57838401 | A | TG | 0.019 | | 0.009 |
| rs11205760 | 1: 51174330 | C | HDL | 0.007 | 0.007 | rs58542926 | 19: 19379549 | C | TG | 0.064 | | 0.021 |
| rs2820443 | 1: 219753509 | C | HDL | 0.009 | 0.003 | rs12463617 | 2: 629244 | C | TG | 0.037 | | 0.011 |
| rs11257655 | 10: 12307894 | T | HDL | 0.013 | 0.011 | rs11125936 | 2: 62871225 | T | TG | 0.025 | | 0.002 |
| rs174547 | 11: 61570783 | T | HDL | 0.046 | 0.000 | rs6093446 | 20: 39780932 | A | TG | 0.032 | | 0.003 |
| rs9568867 | 13: 54107352 | G | HDL | 0.013 | 0.004 | rs738409 | 22: 44324727 | C | TG | 0.039 | | 0.022 |
| rs11847697 | 14: 30515112 | T | HDL | 0.085 | 0.000 | rs888789 | 5: 74988369 | A | TG | 0.013 | | 0.000 |
| rs11634397 | 15: 80432222 | A | HDL | 0.019 | 0.000 | rs832552 | 5: 56113850 | G | TG | 0.016 | | 0.000 |

Abbreviation: BMI, body mass index; BP, blood pressure; EA, effect allele. HDL-C, high-density lipoprotein cholesterol; LDL-C, low-density lipoprotein cholesterol; PRS, polygenic risk score; T2D, type 2 diabetes; TC, total cholesterol; TG, triglycerides.

## Supplementary Table 5. Basic characteristics according to MASLD and advanced fibrosis

| **Variable *** | **No MASLD** | **MASLD** | **MASLD and**  **no advanced fibrosis** | **Advanced fibrosis in MASLD**^†^ |
| --- | --- | --- | --- | --- |
|  | **(n= 8,192)** | **(n=8,885)** | **(n= 7,432)** | **(n= 1,453)** |
| Age (SD), year | 65.8 (9.6) | 64.7 (8.8) | 64.3 (8.8) | 66.3 (8.7) |
| Female, % | 4930 (60.4) | 5977 (67.1) | 5035 (67.6) | 942 (64.7) |
| **Socioeconomic and lifestyle factors** | | |  |  |
| Urban region, % | 2605 (31.7) | 3211 (36.2) | 2755 (37.3) | 456 (31.1) |
| ≥9 years of education, % | 1160 (15.6) | 1436 (14.9) | 1239 (15.3) | 197 (12.8) |
| Household income ≥35,000 RMB/year, % | 5872 (73.0) | 6661 (73.8) | 5591 (74.1) | 1070 (72.2) |
| Ever regular smoking, % |  |  |  |  |
| Male | 2101 (64.6) | 1878 (64.8) | 1546 (64.6) | 332 (65.3) |
| Female | 113 (2.4) | 99 (2.3) | 83 (2.3) | 16 (2.5) |
| Weekly drinking, % |  |  |  |  |
| Male | 903 (28.1) | 897 (30.6) | 756 (30.3) | 141 (31.9) |
| Female | 104 (2.0) | 116 (2.1) | 106 (2.2) | 10 (1.2) |
| Total physical activity (SD), MET-h/d | 16.3 (14.5) | 15.3 (13.5) | 15.5 (13.7) | 14.3 (12.3) |
| Snoring, % | 3675 (45.0) | 5042 (56.9) | 4158 (56.1) | 884 (61.1) |
| **Blood pressure and anthropometry** | | |  |  |
| SBP (SD), mmHg | 130.3 (21.3) | 135.3 (20.7) | 134.9 (20.5) | 137.8 (21.0) |
| DBP (SD), mm Hg | 76.8 (10.5) | 79.3 (10.6) | 79.3 (10.6) | 79.7 (10.8) |
| RPG (SD), mg/dL | 6.2 (2.3) | 6.7 (2.7) | 6.6 (2.6) | 7.4 (3.4) |
| LDL (SD), mg/dL | 3.2 (1.3) | 3.4 (1.3) | 3.4 (1.4) | 3.3 (0.9) |
| Waist circumference (SD), cm | 80.9 (8.3) | 90.4 (8.4) | 90.1 (8.4) | 92.2 (8.4) |
| BMI (SD), kg/m^2^ | 22.5 (2.7) | 26.1 (3.2) | 26.0 (3.1) | 26.8 (3.3) |
| Liver VCTE measurement | | |  |  |
| FAP (IQR), dB/m | 221.0 (26.0) | 272.0 (29.0) | 272.0 (29.0) | 279.0 (31.0) |
| LSM (IQR), kPa | 6.7 (2.8) | 7.0 (3.3) | 6.6 (2.4) | 11.7 (2.6) |
| Advanced fibrosis, % ^†^ | 803 (9.8) | 1453 (16.7) | 0 | 1453 (100.0) |
| **CVH** |  |  |  |  |
| LE8 score (SD) | 65.5 (11.6) | 57.7 (11.6) | 58.1 (11.5) | 55.6 (11.6) |
| LE8 score behavior scale (SD) | 58.5 (17.0) | 56.8 (16.6) | 56.8 (16.6) | 56.4 (16.5) |
| LE8 score biological scale (SD) | 85.9 (18.1) | 70.9 (17.5) | 71.7 (17.4) | 66.7 (17.6) |
| **Prior disease history, %** | |  |  |  |
| Diabetes | 849 (10.4) | 1518 (17.1) | 1169 (15.7) | 349 (23.6) |
| Ischemic heart disease | 604 (7.7) | 854 (9.3) | 665 (8.9) | 189 (11.2) |
| Stroke or TIA | 450 (5.5) | 632 (7.1) | 511 (7.0) | 121 (7.8) |
| Hypertension | 2316 (27.6) | 3718 (42.8) | 2986 (41.7) | 732 (48.4) |
| Family history of diabetes | 957 (12.5) | 1367 (14.7) | 1134 (14.4) | 233 (16.2) |
| Family history of cancer | 1693 (21.5) | 2087 (22.9) | 1801 (23.2) | 286 (21.3) |
| **Medication use, %** |  |  |  |  |
| Statin medication | 288 (3.6) | 475 (5.2) | 382 (5.1) | 93 (5.8) |
| Antihypertensive | 2078 (24.8) | 3426 (39.4) | 2744 (38.3) | 682 (44.9) |
| Diabetes treatment | 647 (7.9) | 1124 (12.7) | 866 (11.8) | 258 (17.0) |
| **Genetic risk category, %** |  |  |  |  |
| Low | 1985 (21.3) | 1364 (17.8) | 1113 (17.9) | 251 (17.2) |
| Intermediate | 4823 (60.8) | 5442 (59.9) | 4536 (59.9) | 906 (59.7) |
| High | 1384 (17.8) | 2079 (22.3) | 1783 (22.1) | 296 (23.2) |

Abbreviations: BMI, body mass index; CVH, cardiovascular health; FAP, fat attenuation parameter; LSM, liver stiffness measurement; MASLD, metabolic dysfunction-associated steatotic liver disease; MET, metabolic equivalent of the task; SBP, systolic blood pressure; TIA, transient ischemic attack; VCTE, vibration-controlled transient elastography.

*Values are means (standard deviation, SD), median (interquartile range, IQR), or n (%). Results were standardized by age, sex, and region where appropriate.

^†^Advanced fibrosis is defined as LSM ≥10.0 kPa.

## Supplementary Table 6. Associations of Life’s Essential 8 with mortality

|  |  | **Model 1** | |  | **Model 2** | |
| --- | --- | --- | --- | --- | --- | --- |
|  | **N. cases/N. total** | **OR (95 CI%)** | ***P-value*** |  | **OR (95 CI%)** | ***P-value*** |
| **Total population** |  |  |  |  |  |  |
| Low CVH | 102/2913 | Reference | |  | Reference | |
| Moderate CVH | 340/13038 | 0.92 (0.73, 1.17) | 0.508 |  | 0.92 (0.72, 1.16) | 0.473 |
| High CVH | 9/1126 | 0.42 (0.21, 0.85) | 0.015 |  | 0.36 (0.17, 0.75) | 0.007 |
| Per SD increment in LE8 | 451/17077 | 0.89 (0.80, 0.98) | 0.019 |  | 0.87 (0.79, 0.97) | 0.009 |
| **MASLD patients** |  |  |  |  |  |  |
| Low CVH | 57/2168 | Reference | |  | Reference | |
| Moderate CVH | 133/6482 | 1.07 (0.77, 1.49) | 0.683 |  | 1.04 (0.74, 1.45) | 0.824 |
| High CVH | 0/235 | NA | |  | NA | |
| Per SD increment in LE8 | 190/8885 | 0.88 (0.75, 1.03) | 0.112 |  | 0.86 (0.74, 1.01) | 0.067 |
| **Non-MASLD participants** |  |  |  |  |  |  |
| Low CVH | 45/745 | Reference | |  | Reference | |
| Moderate CVH | 207/6556 | 0.69 (0.48, 0.98) | 0.039 |  | 0.70 (0.49, 1.00) | 0.050 |
| High CVH | 9/891 | 0.38 (0.18, 0.80) | 0.011 |  | 0.33 (0.15, 0.73) | 0.006 |
| Per SD increment in LE8 | 261/8192 | 0.85 (0.74, 0.97) | 0.019 |  | 0.84 (0.74, 0.97) | 0.015 |

Abbreviations: CI, confidence interval; CVH, cardiovascular health; OR, odds ratio.

CVH was measured by Life’s Essential 8 using the standard scoring algorithm. The unweighted average of all components was then classified as low CVH (0-49 points), moderate CVH (50-79 points), or high CVH (80-100 points).

The multivariable logistics models was used to estimate ORs and 95% CIs. Model 1 was adjusted for age, sex and region, model 2 was additionally adjusted for age, sex, region, marital status, household income and education.

## Supplementary Table 7. Associations of Life’s Crucial 9 with MASLD and advanced Fibrosis in MASLD and linear associations by stratum of genetic risk

|  | **MASLD** | |  | **Advanced fibrosis in MASLD*** | |
| --- | --- | --- | --- | --- | --- |
|  | **OR (95 CI%)** | ***P*-value^†^** |  | **OR (95 CI%)** | ***P*-value^†^** |
| **CVH category** |  |  |  |  |  |
| Low CVH | Reference | |  | Reference | |
| Moderate CVH | 0.28 (0.26, 0.31) | <0.001 |  | 0.20 (0.17, 0.23) | <0.001 |
| High CVH | 0.06 (0.05, 0.08) | <0.001 |  | 0.03 (0.01, 0.05) | <0.001 |
|  |  |  |  |  |  |
| **CVH score, per SD increment** |  |  |  |  |  |
| Overall | 0.45 (0.43, 0.47) | <0.001 |  | 0.37 (0.34, 0.39) | <0.001 |
| High genetic risk | 0.41 (0.38, 0.45) | <0.001 |  | 0.32 (0.27, 0.39) | <0.001 |
| Intermediate genetic risk | 0.46 (0.44, 0.48) | <0.001 |  | 0.37 (0.34, 0.41) | <0.001 |
| Low genetic risk | 0.47 (0.43, 0.51) | <0.001 |  | 0.40 (0.34, 0.47) | <0.001 |
| *P*-value for interaction^‡^ | 0.0297 | |  | <0.001 | |

Abbreviations: CI, confidence interval; CVH, cardiovascular health; MASLD, metabolic dysfunction-associated steatotic liver disease; OR, odds ratio; PRS, polygenetic risk score.

CVH was measured by Life’s Crucial 9, a framework that tentatively extended from the Life's Essential 8 by encompassing psychological health, as endorsed by the American Heart Association.^25^ the multivariable logistics model was used to estimate ORs and 95% CIs. The model was adjusted for age, sex, region, marital status, household income, and education. Analysis was conducted on participants who did not have MASLD as the reference group.

*****Advanced fibrosis is defined as LSM ≥10.0 kPa.

^†^The associations of CVH with MASLD and advanced fibrosis in MASLD were significant when using the Bonferroni-adjusted *p*-value threshold of 0.00556 (0.05/9).

^‡^Interactions were tested using a likelihood ratio test to compare two linear models, including the interaction term of metaPRS and Life’s Crucial 9.

## Supplementary Table 8. Associations between each trait PRS and metaPRS with MASLD and advanced fibrosis in MASLD

|  | **MASLD** | |  | **Advanced fibrosis in MASLD** | |
| --- | --- | --- | --- | --- | --- |
|  | **OR (95 CI%)** | ***P*-value** |  | **OR (95 CI%)** | ***P*-value** |
| **MetaPRS** |  |  |  |  |  |
| Low genetic risk | Reference |  |  | Reference |  |
| Intermediate genetic risk | 1.48 (1.35, 1.63) | 2.18E-16 |  | 1.64 (1.38, 1.96) | 4.02E-08 |
| High genetic risk | 2.01 (1.77, 2.29) | 1.23E-26 |  | 2.24 (1.76, 2.85) | 5.75E-11 |
| Per SD increment in PRS | 1.38 (1.32, 1.45) | 1.70E-43 |  | 1.45 (1.33, 1.58) | 1.01E-17 |
| **NAFLD PRS** |  |  |  |  |  |
| Low genetic risk | Reference |  |  | Reference |  |
| Intermediate genetic risk | 1.07 (0.99, 1.16) | 8.21E-02 |  | 1.13 (0.97, 1.32) | 1.19E-01 |
| High genetic risk | 1.26 (1.14, 1.39) | 3.11E-06 |  | 1.46 (1.22, 1.75) | 3.70E-05 |
| Per SD increment in PRS | 1.08 (1.05, 1.12) | 4.33E-07 |  | 1.15 (1.09, 1.22) | 6.62E-07 |
| **T2D PRS** |  |  |  |  |  |
| Low genetic risk | Reference |  |  | Reference |  |
| Intermediate genetic risk | 1.15 (1.06, 1.24) | 6.81E-04 |  | 1.26 (1.09, 1.47) | 2.53E-03 |
| High genetic risk | 1.31 (1.19, 1.44) | 4.28E-08 |  | 1.45 (1.21, 1.74) | 6.58E-05 |
| Per SD increment in PRS | 1.10 (1.06, 1.13) | 3.05E-09 |  | 1.14 (1.08, 1.21) | 5.23E-06 |
| **BMI PRS** |  |  |  |  |  |
| Low genetic risk | Reference |  |  | Reference |  |
| Intermediate genetic risk | 1.37 (1.26, 1.48) | 1.23E-14 |  | 1.38 (1.18, 1.61) | 4.13E-05 |
| High genetic risk | 1.80 (1.63, 1.98) | 3.65E-32 |  | 1.83 (1.52, 2.20) | 1.14E-10 |
| Per SD increment in PRS | 1.23 (1.19, 1.27) | 2.86E-39 |  | 1.25 (1.18, 1.33) | 2.67E-14 |
| **TC PRS** |  |  |  |  |  |
| Low genetic risk | Reference |  |  | Reference |  |
| Intermediate genetic risk | 0.97 (0.89, 1.05) | 4.13E-01 |  | 0.88 (0.76, 1.02) | 8.54E-02 |
| High genetic risk | 0.89 (0.81, 0.98) | 1.89E-02 |  | 0.81 (0.68, 0.97) | 1.97E-02 |
| Per SD increment in PRS | 0.95 (0.92, 0.98) | 5.50E-04 |  | 0.93 (0.88, 0.98) | 1.04E-02 |
| **LDL PRS** |  |  |  |  |  |
| Low genetic risk | Reference |  |  | Reference |  |
| Intermediate genetic risk | 0.95 (0.87, 1.04) | 2.42E-01 |  | 0.89 (0.76, 1.05) | 1.60E-01 |
| High genetic risk | 0.88 (0.79, 0.98) | 1.66E-02 |  | 0.85 (0.71, 1.03) | 9.52E-02 |
| Per SD increment in PRS | 0.96 (0.93, 0.99) | 6.09E-03 |  | 0.95 (0.90, 1.01) | 1.06E-01 |
| **TG PRS** |  |  |  |  |  |
| Low genetic risk | Reference |  |  | Reference |  |
| Intermediate genetic risk | 0.97 (0.89, 1.05) | 4.16E-01 |  | 0.88 (0.76, 1.01) | 7.53E-02 |
| High genetic risk | 1.01 (0.91, 1.11) | 9.18E-01 |  | 0.84 (0.70, 1.01) | 6.48E-02 |
| Per SD increment in PRS | 1.00 (0.97, 1.04) | 7.91E-01 |  | 0.95 (0.90, 1.01) | 7.66E-02 |
| **HDL PRS** |  |  |  |  |  |
| Low genetic risk | Reference |  |  | Reference |  |
| Intermediate genetic risk | 1.02 (0.94, 1.10) | 6.39E-01 |  | 0.91 (0.79, 1.05) | 2.07E-01 |
| High genetic risk | 0.93 (0.84, 1.02) | 1.42E-01 |  | 0.87 (0.72, 1.04) | 1.25E-01 |
| Per SD increment in PRS | 0.97 (0.94, 1.00) | 4.84E-02 |  | 0.95 (0.90, 1.01) | 7.88E-02 |
| **BP PRS** |  |  |  |  |  |
| Low genetic risk | Reference |  |  | Reference |  |
| Intermediate genetic risk | 1.09 (1.00, 1.18) | 3.99E-02 |  | 1.13 (0.97, 1.31) | 1.26E-01 |
| High genetic risk | 1.13 (1.02, 1.24) | 1.74E-02 |  | 1.18 (0.98, 1.42) | 7.62E-02 |
| Per SD increment in PRS | 1.05 (1.02, 1.08) | 1.45E-03 |  | 1.07 (1.01, 1.14) | 1.85E-02 |

Abbreviations: PRS, polygenetic risk score; LE8, Life's Essential 8; MASLD, metabolic dysfunction-associated steatotic liver disease; NAFLD, Non-alcoholic fatty liver disease; OR, odds ratio. Logistics models were used to estimate ORs and 95% CIs per SD increment of the LE8. Models were adjusted for age, sex, region, and 11 national principal components. The genetic risk was categorized as low (bottom quintile), intermediate (2nd-4th quintile), and high (top quintile) according to quintile cutoff points of the PRS.

## Supplementary Table 9. Associations of LE8 with MASLD and advanced fibrosis in MASLD stratified by and jointly with genetic risk categories (based on metaPRS tertiles)

| **Analysis and Groups^‡^** | **MASLD** | |  | **Advanced fibrosis in MASLD*** | |
| --- | --- | --- | --- | --- | --- |
|  | **OR (95 CI%)** | ***P*-value^†^** |  | **OR (95 CI%)** | ***P*-value^†^** |
| **Stratified analysis** |  |  |  |  |  |
| High genetic risk | 0.42 (0.39, 0.45) | <0.001 |  | 0.32 (0.28, 0.37) | <0.001 |
| Intermediate genetic risk | 0.46 (0.43, 0.49) | <0.001 |  | 0.38 (0.33, 0.43) | <0.001 |
| Low genetic risk | 0.47 (0.44, 0.50) | <0.001 |  | 0.40 (0.35, 0.45) | <0.001 |
|  |  | |  |  | |
| **Joint analysis** |  |  |  |  |  |
| High genetic risk + Low CVH | Reference | |  | Reference | |
| High genetic risk + Moderate CVH | 0.23 (0.18, 0.28) | <0.001 |  | 0.13 (0.10, 0.18) | <0.001 |
| High genetic risk + High CVH | 0.05 (0.04, 0.07) | <0.001 |  | 0.03 (0.02, 0.06) | <0.001 |
| Intermediate genetic risk + Low CVH | 0.61 (0.47, 0.79) | <0.001 |  | 0.53 (0.38, 0.75) | <0.001 |
| Intermediate genetic risk + Moderate CVH | 0.18 (0.15, 0.23) | <0.001 |  | 0.12 (0.09, 0.16) | <0.001 |
| Intermediate genetic risk + High CVH | 0.05 (0.04, 0.07) | <0.001 |  | 0.02 (0.01, 0.04) | <0.001 |
| Low genetic risk + Low CVH | 0.45 (0.35, 0.58) | <0.001 |  | 0.34 (0.24, 0.49) | <0.001 |
| Low genetic risk + Moderate CVH | 0.14 (0.11, 0.18) | <0.001 |  | 0.09 (0.07, 0.13) | <0.001 |
| Low genetic risk + High CVH | 0.02 (0.01, 0.03) | <0.001 |  | 0.01 (0.00, 0.03) | <0.001 |

Abbreviations: CI, confidence interval; CVH, cardiovascular health; LE8, Life’s Essential 8; MASLD, metabolic dysfunction-associated steatotic liver disease; OR, odds ratio.

The multivariable logistics model was used to estimate ORs and 95% Cis, adjusted for age, sex, region, marital status, household income, and education. Analysis was conducted on participants who did not have MASLD as the reference group.

*Advanced fibrosis is defined as LSM ≥10.0 kPa.

†The associations of CVH with MASLD and advanced fibrosis in MASLD were significant when using the Bonferroni-adjusted p-value threshold of 0.00625 (0.05/8).

‡ Stratified analysis: CVH (per SD increment) associated with MASLD and advanced fibrosis within each genetic risk category. Joint analysis: Joint associations of CVH categories (low/moderate/high) and genetic risk categories (low/intermediate/high) with MASLD and advanced fibrosis. Genetic risk categories were defined by metaPRS tertiles (low/intermediate/high)..

## Supplementary Table 10. Sensitivity analysis of associations between LE8 scores with MASLD and fibrosis in MASLD by stratum of metaPRS

|  | **N. cases/N. total** | **Model 1** | |  | **Model 2** | |
| --- | --- | --- | --- | --- | --- | --- |
|  |  | **OR (95 CI%)** | ***P*-value** |  | **OR (95 CI%)** | ***P*-value** |
| **MASLD with moderate/severe liver steatosis, ≥S2** | | |  |  |  |  |
| High genetic risk | 1259/3463 | 0.42 (0.38, 0.46) | <.0001 |  | 0.42 (0.38, 0.45) | <0.001 |
| Intermediate genetic risk | 3127/10265 | 0.42 (0.40, 0.45) | <0.001 |  | 0.42 (0.40, 0.45) | <0.001 |
| Low genetic risk | 721/3349 | 0.45 (0.41, 0.50) | <0.001 |  | 0.46 (0.41, 0.50) | <0.001 |
| ***P*-value for interaction** |  | <0.001 | |  | <0.001 | |
| **MASLD with severe liver steatosis, S3** | |  |  |  |  |  |
| High genetic risk | 354/3463 | 0.42 (0.37, 0.48) | <.001 |  | 0.41 (0.36, 0.47) | <0.001 |
| Intermediate genetic risk | 862/10265 | 0.42 (0.38, 0.45) | <.001 |  | 0.42 (0.38, 0.45) | <0.001 |
| Low genetic risk | 187/3349 | 0.45 (0.38, 0.53) | <.001 |  | 0.45 (0.38, 0.53) | <0.001 |
| ***P*-value for interaction** |  | <0.001 | |  | <0.001 | |
| **Significant fibrosis in MASLD, ≥F2** | |  |  |  |  |  |
| High genetic risk | 751/2135 | 0.36 (0.32, 0.40) | <0.001 |  | 0.35 (0.31, 0.40) | <0.001 |
| Intermediate genetic risk | 2026/6849 | 0.41 (0.38, 0.44) | <0.001 |  | 0.41 (0.38, 0.44) | <0.001 |
| Low genetic risk | 525/2510 | 0.42 (0.38, 0.48) | <0.001 |  | 0.42 (0.37, 0.47) | <0.001 |
| *P*-value for interaction |  | <0.001 | |  | <0.001 | |
| **Cirrhosis in MASLD, F4** |  |  |  |  |  |  |
| High genetic risk | 68/1452 | 0.30 (0.22, 0.41) | <0.001 |  | 0.29 (0.21, 0.41) | <0.001 |
| Intermediate genetic risk | 207/5030 | 0.33 (0.28, 0.40) | <0.001 |  | 0.33 (0.28, 0.39) | <0.001 |
| Low genetic risk | 67/2052 | 0.36 (0.27, 0.48) | <0.001 |  | 0.35 (0.26, 0.47) | <0.001 |
| ***P*-value for interaction** |  | <0.001 | |  | <0.001 | |

Abbreviations: CI, confidence interval; FAP, fat attenuation parameter; LSM, liver stiffness measurement; PRS, polygenetic risk score; LE8, Life's Essential 8; MASLD, metabolic dysfunction-associated steatotic liver disease; OR, odds ratio.

FAP values ≥269 dB/m, and 296 dB/m indicated moderate, and severe liver steatosis (grade S2, and S3, respectively). LSM values ≥7.85 kPa, and 13.5 kPa indicated significant fibrosis and cirrhosis (F2 and F4, respectively). Logistics models were used to estimate ORs and 95% CIs per SD increment of LE8. Model 1 was adjusted for age, sex, and region, model 2 was additionally adjusted for marital status, household income, and education. The genetic risk was categorized high (top quintile), intermediate (2nd-4th quintiles), and low (bottom quintile) according to quintile cut-off points of metaPRS. Interactions were tested using a likelihood ratio test to compare the linear models including the interaction term or not.

## Supplementary Table 11. Associations of LE8 with MASLD and advanced fibrosis in MASLD in subgroup analysis

|  | **MASLD** | |  | **Advanced Fibrosis in MASLD** | |
| --- | --- | --- | --- | --- | --- |
|  | **OR (95 CI%)** | ***P-*value** |  | **OR (95 CI%)** | ***P-*value** |
| **Sex** |  |  |  |  |  |
| **Male** |  |  |  |  |  |
| Low CVH | Reference | |  | Reference | |
| Moderate CVH | 0.28 (0.24, 0.31) | <0.001 |  | 0.22 (0.18, 0.28) | <0.001 |
| High CVH | 0.06 (0.04, 0.09) | <0.001 |  | 0.02 (0.00, 0.08) | <0.001 |
| Per SD increment | 0.46 (0.44, 0.49) | <0.001 |  | 0.40 (0.36, 0.45) | <0.001 |
| **Female** |  |  |  |  |  |
| Low CVH | Reference | |  | Reference | |
| Moderate CVH | 0.30 (0.26, 0.34) | <0.001 |  | 0.19 (0.16, 0.24) | <0.001 |
| High CVH | 0.07 (0.06, 0.09) | <0.001 |  | 0.04 (0.02, 0.06) | <0.001 |
| Per SD increment | 0.44 (0.42, 0.46) | <0.001 |  | 0.34 (0.31, 0.38) | <0.001 |
| ***P*-value for interaction** | 0.028 | |  | <0.001 | |
| **Age** |  |  |  |  |  |
| **<65** |  |  |  |  |  |
| Low CVH | Reference | |  | Reference | |
| Moderate CVH | 0.24 (0.21, 0.29) | <0.001 |  | 0.14 (0.11, 0.19) | <0.001 |
| High CVH | 0.06 (0.05, 0.08) | <0.001 |  | 0.03 (0.02, 0.05) | <0.001 |
| Per SD increment | 0.44 (0.41, 0.46) | <0.001 |  | 0.32 (0.29, 0.36) | <0.001 |
| **≥65** |  |  |  |  |  |
| Low CVH | Reference | |  | Reference | |
| Moderate CVH | 0.32 (0.28, 0.36) | <0.001 |  | 0.25 (0.21, 0.30) | <0.001 |
| High CVH | 0.06 (0.05, 0.09) | <0.001 |  | 0.04 (0.02, 0.08) | <0.001 |
| Per SD increment | 0.46 (0.44, 0.49) | <0.001 |  | 0.40 (0.36, 0.43) | <0.001 |
| ***P*-value for interaction** | 0.006 | |  | 0.229 | |
| **Region** |  |  |  |  |  |
| **Urban** |  |  |  |  |  |
| Low CVH | Reference | |  | Reference | |
| Moderate CVH | 0.33 (0.28, 0.39) | <0.001 |  | 0.23 (0.18, 0.30) | <0.001 |
| High CVH | 0.08 (0.06, 0.10) | <0.001 |  | 0.01 (0.00, 0.05) | <0.001 |
| Per SD increment | 0.47 (0.44, 0.50) | <0.001 |  | 0.36 (0.32, 0.42) | <0.001 |
| **Rural** |  |  |  |  |  |
| Low CVH | Reference | |  | Reference | |
| Moderate CVH | 0.27 (0.24, 0.30) | <0.001 |  | 0.19 (0.16, 0.23) | <0.001 |
| High CVH | 0.06 (0.05, 0.08) | <0.001 |  | 0.04 (0.03, 0.06) | <0.001 |
| Per SD increment | 0.44 (0.42, 0.46) | <0.001 |  | 0.36 (0.33, 0.40) | <0.001 |
| ***P*-value for interaction** | 0.520 | |  | 0.954 | |

Abbreviations: CI, confidence interval; CVH, cardiovascular health; MASLD, metabolic dysfunction-associated steatotic liver disease; OR, odds ratio.

Advanced fibrosis is defined as LSM ≥10.0 kPa. The associations of CVH with MASLD and advanced fibrosis were significant using the Bonferroni-adjusted *p*-value threshold of 0.00625 (0.05/8). Logistics models were used to estimate ORs and 95% CIs. Models were adjusted for age, sex, region, marital status, household income, and education.

## Supplementary Table 12. The associations of genetic risk with NAFLD, advanced severe liver diseases in previous studies

| **Studies** | **Study population** | **N. total** | **N. variants** | **Outcomes** | **Outcome measurement** | **ORs (95% CIs)** | **Genetic risk metrics** |
| --- | --- | --- | --- | --- | --- | --- | --- |
| Copenhagen, Denmark,  UK Biobank^34^ | Predominant European, multi-ethnic | 445,452 | 3 | Cirrhosis (n=817) | ICD10 | 1.6 (1.3-1.9) | 1 scores vs 0 score |
|  |  |  |  | HCC (n=204) |  | 1.2 (0.9-1.7) | 1 scores vs 0 score |
| UK Biobank^35^ | Predominant European, multi-ethnic | 266,687 | 4 | Severe liver diseases (n=543) | ICD-10 | 1.6 (1.3-2.1) ^†^ | Top 25% vs Bottom 10% |
| Michigan Genomics Initiative^36^ | European | 51,550 | 17 | NAFLD (n=3,021) | ICD-9 or  ICD-10 | 2.8 (2.4-3.3) | Top 10% vs Bottom 10% |
|  |  |  |  | Cirrhosis (n=1,472) |  | 2.5 (2.0-3.2) |  |
|  |  |  |  | HCC (n=295) |  | 2.9 (1.8-4.8) |  |
| Partners HealthCare Biobank^37^ | Predominant European, multi-ethnic | 30,469 | 12 | Cirrhosis (n=1,137) | ICD and biopsy-proven | 2.3 (1.9-2.7) | Top 20% vs Bottom 20% |
| Tertiary care medical centers in Austria, Switzerland and Germany^38^ | European | 703 NAFLD patients | 3 | Fibrosis in NAFLD (n=81) | Biopsy-proven | 2.3 (1.0-5.2) | 2 risk alleles vs 0-1 risk alleles |
|  |  |  |  |  |  | 2.6 (1.1-6.0) | 3 risk alleles vs 0-1 risk alleles |
| DHS, Dallas Biobank, CCHS, and CGPS^39^ | Multi-ethnic | 101,828 | 1 | Cirrhosis | ICD-10 | 1.5 (1.2-1.9) | 1 risk alleles vs 0 risk alleles |
|  |  |  |  |  |  | 3.2 (2.2-4.3) | 2 risk alleles vs 0 risk alleles |
| UK biobank^40^ | Predominant European, multi-ethnic | 101,828 | 55 | NAFLD (n=2,274) | ICD-9 and ICD-10 | 1.3 (1.2-1.4) * | Second tertile vs First tertile |
|  |  |  |  |  |  | 1.7 (1.6-1.9) + | Third tertile vs First tertile |

Abbreviations: CCHS, the Copenhagen City Heart Study; CGPS, the Copenhagen General Population Study; DHS, the Dallas Heart Study; ICD-10, International Statistical Classification of Diseases and Related Health Problems, revision 10; HCC, Hepatocellular carcinoma; metaPRS, combined polygenetic risk score; NAFLD, non-alcoholic fatty liver diseases.

^†^ The comparable OR of genetic risk scores in this study for the top 25% versus the bottom 10% metaPRS, was 1.87 (95% CI, 1.45-2.41).

* The comparable OR of genetic risk scores in this study for the second tertile versus first tertile metaPRS, was 1.35 (95% CI, 1.24, 1.47).

+ The comparable OR of genetic risk scores in this study for the third tertile versus first tertile metaPRS, was 1.74 (95% CI, 1.59, 1.92).

## Supplementary Table 13. Interaction analysis of genetic factors and CVH in previous studies on NAFLD and advanced severe liver diseases

| **Studies** | **Study population** | **N. cases/**  **N. total** | **Exposures** | **Outcomes** | **Liver fat measurement** | **N. variants** | ***p*-value of interaction** | **Effect sizes** | **Metrics** |
| --- | --- | --- | --- | --- | --- | --- | --- | --- | --- |
| UK biobank^40^ | Predominant European, multi-ethnic | 2,284/266,645 | LE8 | Severe NAFLD | ICD | 55 | 0.835 | 0.38 vs 0.40 | HR in high genetic risk score vs  HR in low genetic risk score |
| Framingham Heart Study^41^ | US adults | 479/2,773 | LS7 | NAFLD | CT scan | 5 | 0.04 | -0.006 vs -0.011 vs -0.012 | β in lowest tertile of GRS vs  β in intermediate tertile of GRS vs β in high tertile of GRS |
| Framingham Heart Study^42^ | US adults | 1,521 | Diet | NAFLD | CT scan | 5 | <0.001 | Liver fat change was significant only in participants with the lowest-tertile category of dietary score change | |
| Copenhagen, Denmark,UK Biobank^34^ | European | 445,452 | BMI | ALT | Standard hospital assays | 3 | <.0001 | 61% vs 12% | Relative different in BMI >35kg/m^2^ vs in BMI <25 kg/m^2^ population |
|  |  |  | Diabetes |  |  |  | <.0001 |  |  |
| Copenhagen Cohort^39^ | Multi-ethnic | 33,603/93,719 | BMI | Hepatic steatosis | Proton magnetic resonance spectrometry (HTGC >=5.5%) | 1 | 0.028 to 8.7x10^-10^ | 84% vs 42% | Probability of cirrhosis of 2 risk alleles participants in obesity population vs  0 risk alleles in obesity population |
|  |  | 384/93,719 |  | Cirrhosis | ICD |  | 0.026 | 5.8 vs 2.4 | OR in BMI >35 kg/m^2^ population vs OR in BMI <25 kg/m^2^ population |
| Partners HealthCare Biobank^37^ | Predominant European, multi-ethnic | 1,137/30,469 | BMI | Cirrhosis | physician-coded etiologic | 12 | <0.001 | 19.5% vs 10.3% vs 6.5% | Probability of cirrhosis in obesity vs in overweight vs normal weight population |
| UK Biobank^35^ | Predominant European, multi-ethnic | 543/266,687 | Diabetes | Severe liver diseases | ICD | 4 | NA | 3.24 vs 1.63 | HR in diabetes population vs  HR in overall population |
|  |  |  | Obesity |  |  |  |  | 2.29 vs 1.63 | HR in obesity population vs  HR in overall population |

Abbreviations: ALT, alanine aminotransferase; CAP, controled attenuation parameter; CVH, cardiovascular health; FLI, fatty liver index; CT, computed tomography; LS7, Life’s Simple 7; NAFLD, non-alcoholic fatty liver diseases; US, United states.

## Supplementary Table 14. Associations of CVH with NAFLD by stratum of metaPRS in previous studies

|  | **Framingham Heart Study**^41^ |  | **UKB**^40^ |
| --- | --- | --- | --- |
|  | β (95 CI%) * |  | OR (95 CI%) ^#^ |
| **Genetic risk categorical** |  |  |  |
| High genetic risk | -0.001 (-0.009, -0.015) |  | 0.40 (0.35, 0.46) |
| Intermediate genetic risk | -0.011 (-0.008, -0.013) |  | 0.40 (0.34, 0.47) |
| Low genetic risk | -0.006 (-0.004, -0.008) |  | 0.38 (0.32,0.45) |
| ***P*-value for interaction** | 0.040 |  | 0.835 |

Abbreviations: CI, confidence interval; CVH, cardiovascular health; NAFLD, non-alcohol fatty liver disease; OR, odds ratio.

* Stratified analyses of CVH categories (per 1-unit increment) based on Life’s Simple 7 with liver fat by tertiles of the GRS based on 5 single-nucleotide polymorphisms (SNPs) in the Framingham Heart Study.

^#^ Stratified analyses of CVH categories (per category increment) based on Life’s Essential 8 with new-onset NAFLD, by tertiles of the genetic risk scores based on 55 SNPs, adjusted for age, sex, race, education levels, Townsend deprivation index, alcohol intake, use of antidiabetic drugs and cholesterol-lowering drugs, in UK biobank study.

## Supplementary Table 15. The comparison of diagnostic performance of FibroTouch and FibroScan with liver biopsy

| **Outcomes*** | **Population** | **Outcome degrees** | **Se** | **Sp** | **AUC** |
| --- | --- | --- | --- | --- | --- |
| **FibroTouch vs liver biopsy** | |  |  |  |  |
| Steatosis | 237 multi-center NAFLD patients, 18-65 years, liver biopsy within 2 weeks of FibroTouch examination^51^ | ≥S1 | 0.79 | 0.86 | 0.88 (0.84-0.92) |
|  |  | ≥S2 | 0.87 | 0.90 | 0.93 (0.89-0.97) |
|  |  | S3 | 0.89 | 0.83 | 0.88 (0.81–0.94) |
|  | 85 MAFLD patients combined with T2D, 24-74 years^52^ | ≥S1 | 0.83 | 0.80 | 0.84 (0.67-1.01) |
|  |  | ≥S2 | 0.79 | 0.87 | 0.88 (0.81–0.95) |
|  |  | S3 | 1.00 | 0.71 | 0.89 (0.82–0.95) |
|  | 435 NAFLD patients^53^ | ≥S1 | 0.84 | 0.83 | 0.89 (0.84–0.93) |
|  |  | ≥S2 | 0.90 | 0.41 | 0.76 (0.72–0.81) |
|  |  | S3 | 0.51 | 0.80 | 0.69 (0.62–0.76) |
| Fibrosis | 237 multi-center NAFLD patients, 18-65 years, liver biopsy within 2 weeks of FT examination^51^ | ≥F2 | 0.58 | 0.82 | 0.71 (0.63–0.80) |
|  |  | ≥F3 | 0.68 | 0.72 | 0.71 (0.62–0.80) |
|  |  | F4 | 0.80 | 0.71 | 0.77 (0.58–0.97) |
|  | 227 chronic hepatic liver disease patients^52^ | ≥F2 | 0.85 | 0.53 | 0.78 (0.67-0.83) |
|  |  | ≥F3 | 1.00 | 0.77 | 0.92 (0.85-0.96) |
|  |  | F4 | 0.69 | 0.97 | 0.90 (0.81-0.96) |
|  | 435 CLD patients,undergoing a liver biopsy, FibroScan and FibroTouch^54^ | ≥F2 | 0.52 | 0.90 | 0.84 (0.80-0.88) |
|  |  | ≥F3 | 0.71 | 0.90 | 0.92 (0.89-0.95) |
|  |  | F4 | 0.66 | 0.90 | 0.93 (0.90-0.96) |
|  | 85 MAFLD patients combined with T2D, 24–74 years^52^ | ≥F2 | 0.68 | 0.78 | 0.76 (0.66–0.86) |
|  |  | ≥F3 | 0.72 | 0.78 | 0.81 (0.71–0.91) |
|  |  | F4 | 0.86 | 0.90 | 0.92 (0.85–1.00) |
| **FibroScan vs liver biopsy** | | | | | |
| Steatosis | 404 NAFLD patients,19-77 years, underwent liver biopsy within 2 weeks of FibroScan^55^ | ≥S1 | 0.80 | 0.83 | 0.87 (0.82–0.92) |
|  |  | ≥S2 | 0.70 | 0.76 | 0.77 (0.71–0.82) |
|  |  | S3 | 0.72 | 0.63 | 0.70 (0.64–0.75) |
|  | 393 multi-center cohort of American adults with biopsy proven NAFLD^56^ | ≥S1 | 0.80 | 0.77 | 0.76 (0.64–0.89) |
|  |  | ≥S2 | 0.77 | 0.57 | 0.70 (0.64–0.75) |
|  |  | S3 | 0.80 | 0.40 | 0.58 (0.51–0.64) |
|  | 2723 patients by meta-analysis^54^ | ≥S1 | 0.69 | 0.82 | 0.82 (0.81–0.84) |
|  |  | ≥S2 | 0.77 | 0.81 | 0.87 (0.85–0.88) |
|  |  | S3 | 0.88 | 0.78 | 0.88 (0.86–0.91) |
|  | 930 NAFLD patients^57^ | ≥S1 | 0.80 | 0.74 | 0.82 (0.77-0.87) |
|  |  | ≥S2 | 0.78 | 0.63 | 0.75 (0.72-0.79) |
|  |  | S3 | 0.76 | 0.60 | 0.72 (0.68-0.75) |
| Fibrosis | 393 multi-center cohort of American adults with biopsy proven NAFLD^56^ | ≥F2 | 0.66 | 0.80 | 0.79 (0.74–0.83) |
|  |  | ≥F3 | 0.80 | 0.74 | 0.83 (0.79–0.87) |
|  |  | F4 | 0.89 | 0.86 | 0.93 (0.90–0.97) |
|  | 404 NAFLD patients,19-77 years, underwent liver biopsy within 2 weeks of FibroScan^55^ | ≥F2 | 0.71 | 0.70 | 0.77 (0.72-0.82) |
|  |  | ≥F3 | 0.71 | 0.75 | 0.80 (0.75–0.84) |
|  |  | F4 | 0.85 | 0.79 | 0.89 (0.84–0.93) |
|  | 435 CLD patients,undergoing a liver biopsy, FibroScan and FibroTouch^58^ | ≥F2 | 0.55 | 0.90 | 0.86 (0.82–0.90) |
|  |  | ≥F3 | 0.68 | 0.90 | 0.91 (0.87–0.94) |
|  |  | F4 | 0.66 | 0.90 | 0.96 (0.92–0.98) |

Abbreviations: NAFLD, nonalcoholic fatty liver disease; MAFLD, matobolic fatty liver disease; CLD, chronic liver diseases; T2D, type 2 diabetes; Se, sensitivity; Sp, specificity; AUC, areas under the curve.

* UAP (FibroTouch) cut-offs for steatosis ≥S1, ≥S2, S3 were ranging from 244-295, 269-314, 296-324 dB/m, respectively; CAP (FibroScan) cut-offs for steatosis ≥S1, ≥S2, S3 were ranging from 248-302, 268-331, 280-337 dB/m, respectively; LSM (FibroTouch) cut-offs for fibrosis ≥F2, ≥F3, F4 were ranging from 6.20-10.8, 9.0-13.8, 11.0-20.1 kPa, respectively; LSM (FibroScan) cut-offs for fibrosis ≥F2, ≥F3, F4 were ranging from 8.2-10.0, 8.6-11.8, 13.1-16.5 kPa, respectively.

## Supplementary Table 16. The comparison of FibroTouch and FibroScan attenuation parameter and liver stiffness measurement

| **Population** | **Outcomes** | **variable** | **Correlation** | ***p­*-value** |
| --- | --- | --- | --- | --- |
| 163 patients with chronic liver disease^54^ | Steatosis | FAP | 0.76 | <0.001 |
| 1621 patients with chronic hepatitis, liver fibrosis and healthy volunteers^59^ | Steatosis | FAP | 0.64 | <0.001 |
| 163 patients with chronic liver disease^54^ | Fibrosis | LSM | 0.70 | <0.01 |
| 1621 patients with chronic hepatitis, liver fibrosis and healthy volunteers^59^ | Fibrosis | LSM | 0.62 | <0.01 |
| 435 CLD patients,undergoing a liver biopsy and to receive LS measurements with both the FibroScan and FibroTouch^58^ | Fibrosis | LSM | 0.85 | <0.001 |

Abbreviations: FAP, Fat attenuation parameter; LSM, liver stiffness measurement.

## Supplementary Table 17. Comparison of the prevalence of MASLD in this study with previous comparable studies with mean or median age ranging from 40 to 65

| **Study source** | **No. participants** | **Study years** | **Age** | **Prevalence^‡^** | **Study region** |
| --- | --- | --- | --- | --- | --- |
| CKB (this study)* | 17,077 | 2020-2021 | 65 (45-95)^Ψ^ | MASLD(≥S1) 52.3% | 10 cites (5 rural and 5 urban) in China |
| Shanghai Nicheng Cohort Study^43^** | 6,873 | 2013-2018 | 62 (55-70) ^Ψ^ | MAFLD: 46.7%  Fatty liver disease: 47.2% | Shanghai, China |
| Meinian health check-up^44^* | 5,757,335 | 2017-2022 | 44 (18-≥70) ^Ψ^ | Liver steatosis (≥S1): 44.39% | 30 provinces in China |
| Meinian health check-up^45^** | 13,839,860 | 2019 | 40 (18-≥80) ^Ψ^ | Fatty liver disease: 34.9% | 31 provinces of China |
| Urban population in Xuzhou, regular health check-ups at the Affiliated Hospital of Xuzhou Medical University^46^** | 75,570 | 2021-2022 | 45 (18-≥80) ^Ψ^ | MAFLD: 37.32% | Xuzhou, China |
| 13 sites health examination population in China^47^** | 149,469 | 2011-2017 | 50.8 (10.7) ^+^ | MAFLD: 51.68% | 13 health management centers in China |
| Health Management Center of Tianjin Union Medical Center^48^** | 16,924 | 2020 | 57.2(12.7) ^+^ | MAFLD: 40.76% | Tianjin, China |
| Kailuan study^49^** | 152,139 | 2006-2012 | 51.9 (15.4) ^+^ | MAFLD: 31.5% | Tangshan, Hebei Province, China |
| MJ Health Check-up Study^50^** | 4,524 | 2009-2018 | 45.3 (7.0) ^+^ | MAFLD: 43.4% | Beijing, China |

* The hepatic steatosis diagnosis by transient elastography examination FibroTouch, FAP values ≥244 Db/m, ≥269 dB/m, and ≥296 dB/m indicated mild, moderate, and severe liver steatosis (grade ≥S1, ≥S2, and S3, respectively). ** The diagnosis of hepatic steatosis, confirmed through abdominal ultrasound. ΨThe median (range) of age was shown. ^+^The mean (SD) of age was shown. ‡ MASLD was diagnosed based on liver steatosis, and the presence of any of five cardiometabolic conditions; MAFLD was diagnosed as the presence of fatty liver with ≥1 of the following: (1) overweight or obese (body mass index [BMI] ≥23 kg/m2), (2) diabetes mellitus and (3) at least 2 metabolic risk abnormalities. NAFLD was the presence of fatty liver in the absence of excessive alcohol consumption (<20 g/day for females and <30 g/day for males) or other identifiable causes.

## Supplementary Figure 1. Flow chart of the study

^†^ For participants without liver ultrasound data due to database missing matching issues or emergency (the subject had scoliosis and cannot lie on his back, or the subject had a heart stent, or the device failed to upload the data).

*Contraindication for liver ultrasound test included unhealed wounds in the liver area, severe ascites, implantable devices, metal stents and pregnant.

^#^Valid liver ultrasound measurement: IQR/median ≤ 33%, success number ≥10, and success rate > 60%.

## Supplementary Figure 2. MetaPRS flow diagram

 Abbreviation: PRS, polygenetic risk score.

## Supplementary Figure 3. Correlation plot of NAFLD and related trait-specific PRS

* was marked the correlations which were not significant (significant threshold *p*-value <0.05)

Correlation coefficients and *p-*values were estimated from Pearson correlation test for each pair of PRS.

Abbreviations: PRS, polygenic risk score; BP, blood pressure; BMI, body mass index; HDL-C, high-density lipoprotein cholesterol; LDL-C, low-density lipoprotein cholesterol; T2D, type 2 diabetes; TC, total cholesterol; TG, triglycerides.

## Supplementary Figure 4. Distribution of overall Life's Essential 8 score and each component of Life's Essential 8

The distribution of the LE8 smoking metric was presented for the men participants, as a high proportion of females with high health metrics engaging in smoking (97.1%). Abbreviation: BP, blood pressure；LE8, Life's Essential 8; MASLD, metabolic dysfunction-associated steatotic liver disease.

## Supplementary Figure 5. Dose-response relationships between Life's Essential 8 scores and (A) MASLD and (B) advanced fibrosis in MASLD

Solid lines represent ORs, and the shaded areas represent 95% CIs. Restricted cubic spline regressions with five knots placed at the 5th, 25th, 50th, 75th, and 95th percentiles were used. The models were adjusted for age, sex, region, marital status, household income, and education. The genetic risk was categorized as high (top quintile), intermediate (2nd-4th quintiles), and low (bottom quintile) according to quartile cut-off points of the metaPRS. Abbreviation: PRS, polygenetic risk score; LE8, Life's Essential 8; MASLD, metabolic dysfunction-associated steatotic liver disease.

## Supplementary Figure 6. Association of each component of Life's Essential 8 metrics and psychological health with MASLD and advanced fibrosis in MASLD

ORs (95% CIs) per SD increase in scores of each component of LE8 metrics were obtained by logistics model adjusted for age, sex, region, marital status, household income and education. The association of each individual component of the LE8 was examined with all 8 metrics simultaneously included in the model.

Abbreviations: BMI, body mass index; BP, blood pressure; MASLD, metabolic dysfunction-associated steatotic liver disease; PA, physical activity.

## Supplementary Figure 7. Associations of trait-specific PRS and metaPRS with MASLD

ORs (95% CIs) per SD increase in trait-specific PRS and metaPRS were obtained by logistics model adjusted for age, sex, region and 11 principal components.

Abbreviations: BMI, body mass index; BP, blood pressure; HDL, high-density lipoprotein cholesterol; LDL, low-density lipoprotein cholesterol; T2D, type 2 diabetes; TC, total cholesterol; TG, triglycerides; MASLD, metabolic dysfunction-associated steatotic liver disease; PRS, polygenetic risk score.

## Supplementary Figure 8. Prevalence of MASLD by quintiles of metaPRS and each traits-specific PRS

Abbreviations: PRS, polygenic risk score; BP, blood pressure; BMI, body mass index; HDL, high-density lipoprotein cholesterol; LDL, low-density lipoprotein cholesterol; T2D, type 2 diabetes; TC, total cholesterol; TG, triglycerides.

Reference

1.	Li JJ, Zhao SP, Zhao D, et al. 2023 China Guidelines for Lipid Management. J Geriatr Cardiol 2023;20:621-663.

2.	Clarke R, Wright N, Walters R, et al. Genetically Predicted Differences in Systolic Blood Pressure and Risk of Cardiovascular and Noncardiovascular Diseases: A Mendelian Randomization Study in Chinese Adults. Hypertension 2023;80:566-576.

3.	Evangelou E, Warren HR, Mosen-Ansorena D, et al. Genetic analysis of over 1 million people identifies 535 new loci associated with blood pressure traits. Nat Genet 2018;50:1412-1425.

4.	Visseren FLJ, Mach F, Smulders YM, et al. 2021 ESC Guidelines on cardiovascular disease prevention in clinical practice. Eur Heart J 2021;42:3227-3337.

5.	Du H, Bennett D, Li L, et al. Physical activity and sedentary leisure time and their associations with BMI, waist circumference, and percentage body fat in 0.5 million adults: the China Kadoorie Biobank study. Am J Clin Nutr 2013;97:487-496.

6.	Du H, Li L, Whitlock G, et al. Patterns and socio-demographic correlates of domain-specific physical activities and their associations with adiposity in the China Kadoorie Biobank study. BMC Public Health 2014;14:826.

7.	Wareham NJ, Jakes RW, Rennie KL, Mitchell J, Hennings S, Day NE. Validity and repeatability of the EPIC-Norfolk Physical Activity Questionnaire. Int J Epidemiol 2002;31:168-174.

8.	Matthews CE, Shu XO, Yang G, et al. Reproducibility and validity of the Shanghai Women’s Health Study physical activity questionnaire. Am J Epidemiol 2003;158:1114-1122.

9.	Ainsworth BE, Haskell WL, Herrmann SD, et al. 2011 Compendium of Physical Activities: a second update of codes and MET values. Med Sci Sports Exerc 2011;43:1575-1581.

10.	Bennett DA, Du H, Clarke R, et al. Association of Physical Activity With Risk of Major Cardiovascular Diseases in Chinese Men and Women. JAMA Cardiol 2017;2:1349-1358.

11.	Pang Y. Assessing the Associations of Metabolic Risk Factors with Risk of Cancer in Chinese Adults: A Prospective Study of 0.5 Million People. PhD Thesis. University of Oxford; 2018.

12.	Pols MA, Peeters PH, Ocké MC, Slimani N, Bueno-de-Mesquita HB, Collette HJ. Estimation of reproducibility and relative validity of the questions included in the EPIC Physical Activity Questionnaire. Int J Epidemiol 1997;26 Suppl 1:S181-189.

13.	Hirshkowitz M, Whiton K, Albert SM, et al. National Sleep Foundation’s sleep time duration recommendations: methodology and results summary. Sleep Health 2015;1:40-43.

14.	Diagnostic and Statistical Manual of Mental Disorders: DSM-5 (5th edition). Reference Reviews 2014;28:36-37.

15.	Zhu Y, Fan J, Lv J, et al. Maintaining healthy sleep patterns and frailty transitions: a prospective Chinese study. BMC Med 2022;20:354.

16.	DiBello JR, Kraft P, McGarvey ST, Goldberg R, Campos H, Baylin A. Comparison of 3 methods for identifying dietary patterns associated with risk of disease. Am J Epidemiol 2008;168:1433-1443.

17.	Qin C, Lv J, Yu C, et al. Dietary patterns and cardiometabolic diseases in 0.5 million Chinese adults: a 10-year cohort study. Nutr J 2021;20:74.

18.	Yu C, Shi Z, Lv J, et al. Major Dietary Patterns in Relation to General and Central Obesity among Chinese Adults. Nutrients 2015;7:5834-5849.

19.	Qin C, Guo Y, Pei P, et al. The Relative Validity and Reproducibility of Food Frequency Questionnaires in the China Kadoorie Biobank Study. Nutrients 2022;14:794.

20.	Chen Z, Chen J, Collins R, et al. China Kadoorie Biobank of 0.5 million people: survey methods, baseline characteristics and long-term follow-up. Int J Epidemiol 2011;40:1652-1666.

21.	Santini ZI, Jose PE, Cornwell EY, et al. Social disconnectedness, perceived isolation, and symptoms of depression and anxiety among older Americans (NSHAP): a longitudinal mediation analysis. The Lancet Public Health 2020;5:e62-e70.

22.	Cornwell EY, Waite LJ. Social disconnectedness, perceived isolation, and health among older adults. J Health Soc Behav 2009;50:31-48.

23.	Lloyd-Jones DM, Allen NB, Anderson CAM, et al. Life’s Essential 8: Updating and Enhancing the American Heart Association’s Construct of Cardiovascular Health: A Presidential Advisory From the American Heart Association. Circulation 2022;146:e18-e43.

24.	Levine GN, Cohen BE, Commodore-Mensah Y, et al. Psychological Health, Well-Being, and the Mind-Heart-Body Connection: A Scientific Statement From the American Heart Association. Circulation 2021;143:e763-e783.

25.	Gaffey AE, Rollman BL, Burg MM. Strengthening the Pillars of Cardiovascular Health: Psychological Health is a Crucial Component. Circulation 2024;149:641-643.

26.	Vujkovic M, Ramdas S, Lorenz KM, et al. A multiancestry genome-wide association study of unexplained chronic ALT elevation as a proxy for nonalcoholic fatty liver disease with histological and radiological validation. Nat Genet 2022;54:761-771.

27.	Sveinbjornsson G, Ulfarsson MO, Thorolfsdottir RB, et al. Multiomics study of nonalcoholic fatty liver disease. Nat Genet 2022;54:1652-1663.

28.	Ghodsian N, Abner E, Emdin CA, et al. Electronic health record-based genome-wide meta-analysis provides insights on the genetic architecture of non-alcoholic fatty liver disease. Cell Rep Med 2021;2:100437.

29.	Li J, Tian A, Zhu H, et al. Mendelian Randomization Analysis Reveals No Causal Relationship Between Nonalcoholic Fatty Liver Disease and Severe COVID-19. Clin Gastroenterol H 2022;20:1553-1560.e78.

30.	Xia M, Ma S, Huang Q, et al. NAFLD-related gene polymorphisms and all-cause and cause-specific mortality in an Asian population: the Shanghai Changfeng Study. Aliment Pharm Ther 2022;55:705-721.

31.	Lu X, Liu Z, Cui Q, et al. A polygenic risk score improves risk stratification of coronary artery disease: a large-scale prospective Chinese cohort study. Eur Heart J 2022;43:1702-1711.

32.	Lu X, Niu X, Shen C, et al. Development and Validation of a Polygenic Risk Score for Stroke in the Chinese Population. Neurology 2021;97:e619-e628.

33.	Abraham G, Malik R, Yonova-Doing E, et al. Genomic risk score offers predictive performance comparable to clinical risk factors for ischaemic stroke. Nat Commun 2019;10:5819.

34.	Gellert-Kristensen H, Richardson TG, Davey Smith G, Nordestgaard BG, Tybjaerg-Hansen A, Stender S. Combined Effect of PNPLA3, TM6SF2, and HSD17B13 Variants on Risk of Cirrhosis and Hepatocellular Carcinoma in the General Population. Hepatology 2020;72:845-856.

35.	De Vincentis A, Tavaglione F, Jamialahmadi O, et al. A Polygenic Risk Score to Refine Risk Stratification and Prediction for Severe Liver Disease by Clinical Fibrosis Scores. Clin Gastroenterol Hepatol 2022;20:658-673.

36.	Chen Y, Du X, Kuppa A, et al. Genome-wide association meta-analysis identifies 17 loci associated with nonalcoholic fatty liver disease. Nat Genet 2023;55:1640-1650.

37.	Emdin CA, Haas M, Ajmera V, et al. Association of genetic variation with cirrhosis: a multi-trait genome-wide association and gene-environment interaction study. Gastroenterology 2021;160:1620-1633.e13.

38.	Paternostro R, Staufer K, Traussnigg S, et al. Combined effects of PNPLA3, TM6SF2 and HSD17B13 variants on severity of biopsy-proven non-alcoholic fatty liver disease. Hepatol Int 2021;15:922-933.

39.	Stender S, Kozlitina J, Nordestgaard BG, Tybjærg-Hansen A, Hobbs HH, Cohen JC. Adiposity amplifies the genetic risk of fatty liver disease conferred by multiple loci. Nat Genet 2017;49:842-847.

40.	He P, Zhang Y, Ye Z, et al. A healthy lifestyle, Life’s Essential 8 scores and new-onset severe NAFLD: A prospective analysis in UK Biobank. Metabolism 2023;146:155643.

41.	DeCoste LR, Wang N, Palmisano JN, et al. Adherence to Ideal Cardiovascular Health Metrics Is Associated With Reduced Odds of Hepatic Steatosis. Hepatol Commun 2021;5:74-82.

42.	Ma J, Hennein R, Liu C, et al. Improved Diet Quality Associates With Reduction in Liver Fat, Particularly in Individuals With High Genetic Risk Scores for Nonalcoholic Fatty Liver Disease. Gastroenterology 2018;155:107-117.

43.	Liang Y, Chen H, Liu Y, et al. Association of MAFLD With Diabetes, Chronic Kidney Disease, and Cardiovascular Disease: A 4.6-Year Cohort Study in China. J Clin Endocrinol Metab 2022;107:88-97.

44.	Man S, Deng Y, Ma Y, et al. Prevalence of liver steatosis and fibrosis in the general population and various high-risk populations: a nationwide study with 5.7 million adults in China. Gastroenterology Published online June 26, 2023:S0016-5085(23)00928-9.

45.	K C, Z S, W G, et al. Prevalence of obesity and associated complications in China: A cross-sectional, real-world study in 15.8 million adults. Diabetes, obesity & metabolism 2023;25.

46.	M C, Z S, W W, P S, G S. Sex-specific prevalence and risk factors of metabolic-associated fatty liver disease among 75,570 individuals in eastern China. Frontiers in endocrinology 2023;14.

47.	Lei F, Wang XM, Wang C, et al. Metabolic dysfunction-associated fatty liver disease increased the risk of subclinical carotid atherosclerosis in China. Front Endocrinol (Lausanne) 2023;14:1109673.

48.	Huang YP, Zhang S, Zhang M, et al. Gender-specific prevalence of metabolic-associated fatty liver disease among government employees in Tianjin, China: a cross-sectional study. BMJ Open 2021;11:e056260.

49.	Wang X, Wu S, Yuan X, et al. Metabolic Dysfunction-associated Fatty Liver Disease and Mortality Among Chinese Adults: a Prospective Cohort Study. J Clin Endocrinol Metab 2022;107:e745-e755.

50.	Wang X, Zhang R, Man S, et al. Metabolic-associated fatty liver disease in relation to site-specific and multiple-site subclinical atherosclerosis. Liver Int Published online June 20, 2023.

51.	Qu Y, Song YY, Chen CW, et al. Diagnostic Performance of FibroTouch Ultrasound Attenuation Parameter and Liver Stiffness Measurement in Assessing Hepatic Steatosis and Fibrosis in Patients With Nonalcoholic Fatty Liver Disease. Clin Transl Gastroenterol 2021;12:e00323.

52.	Yu H, Liu H, Zhang J, et al. Accuracy of FibroTouch in assessing liver steatosis and fibrosis in patients with metabolic-associated fatty liver disease combined with type 2 diabetes mellitus. Ann Palliat Med 2021;10:9702-9714.

53.	Zhu SH, Zheng KI, Hu DS, et al. Optimal thresholds for ultrasound attenuation parameter in the evaluation of hepatic steatosis severity: evidence from a cohort of patients with biopsy-proven fatty liver disease. Eur J Gastroenterol Hepatol 2021;33:430-435.

54.	Ng YZ, Lai LL, Wong SW, Mohamad SY, Chuah KH, Chan WK. Attenuation parameter and liver stiffness measurement using FibroTouch vs Fibroscan in patients with chronic liver disease. PLoS One 2021;16:e0250300.

55.	Eddowes PJ, Sasso M, Allison M, et al. Accuracy of FibroScan Controlled Attenuation Parameter and Liver Stiffness Measurement in Assessing Steatosis and Fibrosis in Patients With Nonalcoholic Fatty Liver Disease. Gastroenterology 2019;156:1717-1730.

56.	Siddiqui MS, Vuppalanchi R, Van Natta ML, et al. Vibration-Controlled Transient Elastography to Assess Fibrosis and Steatosis in Patients With Nonalcoholic Fatty Liver Disease. Clin Gastroenterol Hepatol 2019;17:156-163.e2.

57.	Petroff D, Blank V, Newsome PN, et al. Assessment of hepatic steatosis by controlled attenuation parameter using the M and XL probes: an individual patient data meta-analysis. Lancet Gastroenterol Hepatol 2021;6:185-198.

58.	Xu Y, Liu Y, Cao Z, et al. Comparison of FibroTouch and FibroScan for staging fibrosis in chronic liver disease: Single-center prospective study. Dig Liver Dis 2019;51:1323-1329.

59.	Jing Z, Wanlu S, Guangyu C, et al. Efficiency of FibroScan and FibroTouch in liver stiffness measurement and fat quantification: a comparative analysis. Chinese Journal of Hepatology 2016;24:652-658.
